# LEF-6 phosphorylation regulates the binding and trafficking of baculovirus late gene transcripts

**DOI:** 10.1128/jvi.02100-24

**Published:** 2025-08-11

**Authors:** Xiaoyue Zhang, Zhihui Zhang, Ziqian Cao, Jianchao Li, Shengqiang Jiang, Hongying Chen

**Affiliations:** 1 College of Life Sciences, Northwest A&F University 12469, Yangling, Shaanxi, P. R. China; 2 College of Life Sciences, Hebei University 56667, Baoding, Hebei, P. R. China; 3 Institute of Future Agriculture, Northwest A&F University 12469, Yangling, Shaanxi, P. R. China; Wageningen University & Research, Wageningen, Netherlands

**Keywords:** Autographa californica multiple nucleopolyhedrovirus, LEF-6, phosphorylation, RNA binding, mRNA trafficking

## Abstract

The Autographa californica multiple nucleopolyhedrovirus (AcMNPV) LEF-6 has been shown to accelerate the infection cycle of AcMNPV, but its specific function is yet to be defined. In the present study, we identify LEF-6 as an RNA-binding protein that preferentially interacts with viral late transcripts. We demonstrate that the N-terminal half of LEF-6, which contains two typical ribonucleoprotein (RNP) motifs, is responsible for RNA binding, but its C-terminal half is required for the binding specificity to viral RNAs and for fulfilling its function in promoting virus infection. Further investigations reveal that LEF-6 has eight phosphorylated sites, and seven of them are located in the C-terminal half of LEF-6. The phosphorylation of LEF-6, especially the modification on the key site of S95, plays an important role in the binding and trafficking of viral late transcripts into the cytoplasm in baculovirus infection. These findings suggest that LEF-6 facilitates baculovirus infection through recognition and nuclear export of viral late mRNAs for protein translation.

**IMPORTANCE:**

AcMNPV is the most well-studied baculovirus. As the viral vector in baculovirus expression vector system (BEVS), it has been widely used for the production of proteins in the basic research and pharmaceutical industry. In this study, we found that phosphorylated LEF-6 acted as a viral mRNA-binding and trafficking protein to accelerate the translation of viral late proteins and subsequently benefit baculovirus infection. The RNA-binding domain, nuclear localization signal motif, and phosphorylation sites of LEF-6 were determined. Our findings can help elucidate the roles of LEF-6 in baculovirus infectious cycle and improve the BEVS for protein production.

## INTRODUCTION

Baculoviruses are a group of double-stranded DNA viruses with genome sizes ranging from 80 to 180 kb. During infection of insect cells, most baculoviruses undergo a biphasic life cycle with the production of two morphologically distinct but genetically identical virion types: budded virion (BV) and occlusion-derived virion (ODV) (1). One of the most studied baculoviruses is Autographa californica multiple nucleopolyhedrovirus (AcMNPV), a member of species *Alphabaculovirus aucalifornicae* (https://ictv.global/report/chapter/baculoviridae) (2). The gene expression of AcMNPV is under strict temporal control, and viral transcription can be divided into four major phases: immediate early, early, late, and very late phases (3). The expression of late and very late genes is regulated by late expression factors (LEFs) encoded by viral early genes.

By transient expression experiments, 20 late expression factors, namely, LEFs 1–12, IE-1, IE-2, P35, PP31, P47, P143, DNAPOL (4), and VLF-1 (5), have been identified. According to their main functions, LEFs can be divided into two categories: one is replication-related factors that participate in or promote viral DNA replication, and the other one is transcription-related factors that regulate the expression of late genes. Among the 20 *lef* genes, *lef-1*, *lef-2*, *lef-3*, *lef-11*, *ie1*, *p143*, and *dna pol* are essential genes for DNA replication, and *lef-7*, *ie2*, and *p35* play significant roles in promoting DNA replication (6–10). In addition, *pe38* has also been found to activate DNA replication (7, 11, 12). These genes are indirectly involved in the regulation of late gene expression. The remaining 10 *lef* genes can directly regulate the expression of late and very late genes (9, 13). The promoters of late and extremely late genes are recognized and transcribed by the viral-encoded RNA polymerase. The viral RNA polymerase complex consists of four subunits encoded by *lef4*, *lef8*, *lef9*, and *p47* (14). Deletion of *lef-5*, *lef-10*, and *lef-11* dramatically reduces the transcription levels of late genes, and these genes are essential for the production of progeny virions (8, 15, 16). The late transcription factor genes of *lef-6*, *lef-12*, and *pp3*1 are nonessential for BV production (4, 10, 17), but they can facilitate the expression of late genes and promote virion production.

The *lef-6* gene is present in the genomes of all alphabaculoviruses and betabaculoviruses. It was first identified to be involved in the transcription of baculovirus late and very late genes by using a transient expression assay (18). Knockout of *lef-6* in AcMNPV does not affect viral DNA replication, but it results in a substantial delay in the onset of late transcription and reduces viral yields (10). The AcMNPV *lef-6* encodes a predicted protein of 173 amino acids, with a predicted molecular weight of 20.4 kDa. Using HHpred prediction, AcMNPV LEF-6 showed over 80% probability of being related to mRNA export factor TAP (3), which transports mRNA out of the nucleus through interactions with nuclear pore proteins (19, 20). Quantitative phosphoproteome analysis of cells infected with Bombyx mori nuclear polyhedrosis virus (BmNPV) revealed that LEF-6 is highly phosphorylated during virus infection (21). However, it remains unclear whether LEF-6 plays a role in mRNA trafficking and whether the phosphorylation modifications are associated with its function, and the functional motifs of LEF-6 remain largely unexplored.

In this study, we confirmed the reduced effects of *lef-6* gene knockout on AcMNPV late gene expression and virion production. We demonstrated for the first time that LEF-6 preferentially bound to viral late RNAs and assessed the binding affinity of LEF-6 to viral RNA by a fluorescence anisotropy assay. Using mass spectrometry, we identified eight phosphorylation sites on LEF-6. The roles of LEF-6 phosphorylation in late gene expression, RNA binding, and viral late mRNA trafficking were investigated.

## MATERIALS AND METHODS

### Cell lines and vectors

**Fig 1:**
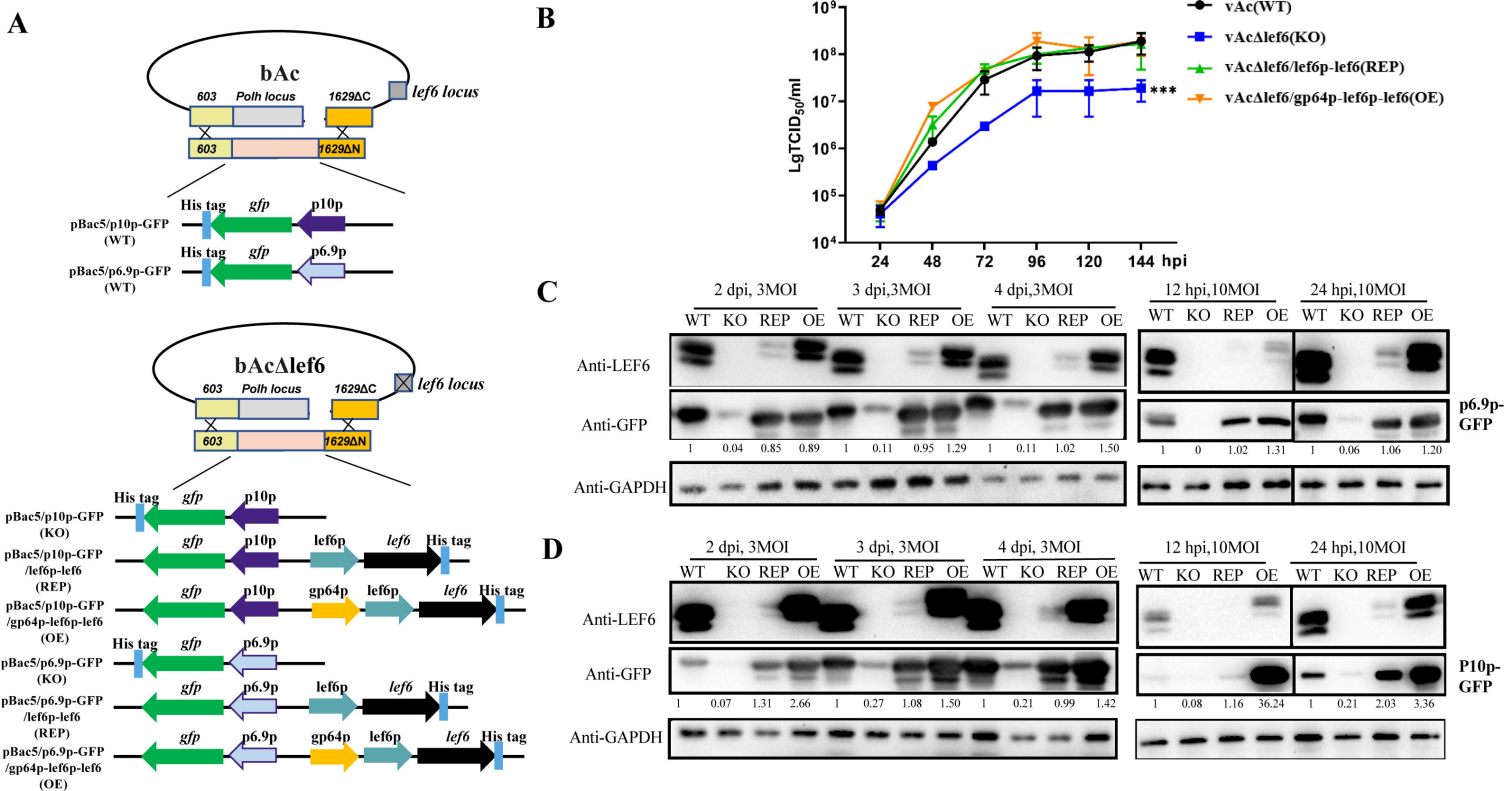
Effects of deletion and complementation of *lef-6* on viral replication and late gene expression. (**A**) Schematic diagram of the generation of wild-type (WT), *lef-6* KO, and restored viruses. The parental bacmid bAc (BAC10:KO1629) was used as a WT control in this study. The *lef-6* KO bacmid, named bAcΔlef6, was constructed by deleting the *lef-6* gene from the bAc bacmid using Red/ET recombination in *E. coli* HS996 strain. Recombinant viruses expressing the reporter gene and the restored *lef-6* at the *polyhedrin* locus were generated in *Sf*9 cells by co-transfection with linearized bAcΔlef6 and the indicated pBac5 plasmids. (**B**) Growth curves of the recombinant viruses. *Sf*9 cells were infected with the indicated recombinant viruses expressing the reporter gene under the control of the *p10* promoter at an MOI of 0.5. Supernatants were collected at 24, 48, 72, 96, 120, and 144 hpi, and the viral titers were determined by an endpoint dilution assay. Data were presented as the means ± SD of three replicates. ***P < 0.001 vs. vAc. (**C and D**) Western blot analysis of protein expression. *Sf*9 cells were infected by the indicated viruses at an MOI of 3 or 10. The GFP reporter was expressed under the control of the viral late gene *p6.9* promoter (**C**) or very late gene *p10* promoter (**D**). The cell lysates harvested at the indicated time were separated by SDS-PAGE and probed by anti-LEF6, anti-GFP, and anti-GAPDH antibody. GAPDH was detected as a protein loading control. The GFP bands were quantified by densitometry scanning using Image J software, and the relative expression levels to the WT are shown below the GFP bands.

*Spodoptera frugiperda* 9 (*Sf*9) cells (Invitrogen) were cultured in SFX insect medium (HyClone, GE Healthcare Life Sciences) supplemented with 1% fetal bovine serum (HyClone) at 27°C. *Trichoplusia ni* (*Tn*) cells (High Five) were maintained in serum-free insect cell culture medium at 27°C. Bacmid BAC10:KO_1629_ (bAc in Fig. 1) was propagated in *E. coli* strain HS996 (22). Plasmids pBac5/p10p-GFP and pTriEx1.1 (Novagen) were stored in our laboratory (23).

### Construction of plasmids

To generate *lef-6* rescue vectors, we modified plasmid pBac5/p10p-GFP. First, a p10p-GFP fragment containing *gfp* under the control of *p10* promoter was amplified from plasmid pBac5/p10p-GFP using primers pBac5-GFP-BglII-F and pBac5-GFP-R. A lef6p-lef6 fragment containing *lef-6* ORF under the control of *lef-6* promoter (−105 to −1 relative to the translation start site) was amplified from bacmid BAC10:KO_1629_ (named as bAc in this study), and then, the fragment p10p-GFP/lef6p-lef6 was obtained by the overlap PCR and inserted into plasmid pBac5/p10p-GFP to replace the *Bgl*II-*Xho*I fragment, generating a modified vector named pBac5/p10p-GFP/lef6p-lef6 (Fig. 1A). To overexpress *lef-6*, the fragment containing *gp64* promoter (−155 to −1 relative to the translation start site) was amplified from bAc, and a p10p-GFP/gp64p-lef6p-lef6 fragment was obtained by overlap PCR so that *lef-6* can be expressed under the tandem promoters of *gp64* and *lef-6*. The fragment was inserted into pBac5/p10p-GFP between the *Bgl*II and *Xho*I sites to generate pBac5/p10p-GFP/gp64p-lef6p-lef6 (Fig. 1A).

To further examine the effect of *lef-6* deletion on late gene expression, the *p10* promoter was replaced with another late gene *p6.9* promoter to control the expression of GFP reporter. The serial plasmids generated were named pBac5/p6.9p-GFP, pBac5/p6.9p-GFP/lef6p-lef6, and pBac5/p6.9p-GFP/gp64p-lef6p-lef6.

All point mutation and truncated mutation fragments were amplified from the wild-type *lef-6* gene using the corresponding PCR primer pairs. All constructed plasmids were confirmed by DNA sequencing. All primers were synthesized by Tsingke Biological Technology. The primer sequences used in this study are listed in Supplementary data Table S1.

### Construction of *lef-6*-knockout bacmids and generation of recombinant baculoviruses

To construct the *lef-6-*knockout bacmid, we removed the *lef-6* gene from the parental bacmid bAc by using the Red/ET recombination system. The rpsL-amp counter-selection cassette with homologous arms containing *lef-6* flanking sequences was amplified by PCR using primers Ac28U and Ac28D. The amplified fragment was transformed into HS996 competent cells containing pSC101-BAD-gbaA and bacmid bAc. Single colonies containing the *lef6*-knockout bacmid bAcΔ*lef6* (rpsL-amp) were screened, and the gene knockout was confirmed by DNA sequencing. In order to remove the rpsL-amp counter-selection cassette, a short double-stranded DNA fragment was obtained by annealing of primers Ac28F and Ac28R and transformed into HS996 competent cells containing pSC101-BAD-gbaA and bAcΔ*lef6* (rpsL-amp). After counter selection, the short fragment replaced the rpsL-amp cassette via homologous recombination, and the resulting bacmid was named bAcΔ*lef6* (Fig. 1A).

To generate *lef6*-knockout recombinant baculovirus, plasmid carrying a reporter gene was co-transfected with the linearized bAcΔ*lef6* into *Sf*9 cells using FuGENE HD Transfection Reagent (Promega). The wild-type virus vAc (WT) was generated as a control by co-transfecting *Sf*9 cells with bacmid BAC10:KO_1629_ (bAc) and the corresponding plasmid (Fig. 1). Supernatant containing the recombinant baculovirus was harvested at 5 days post-transfection (dpt) by centrifugation at 300 × *g* for 5 min to remove cell debris. The viral titer after two passages was determined by 50% tissue culture infective dose (TCID_50_) assay (24).

To examine the homogeneity of the viruses generated by the homologous recombination, DNA was extracted from the supernatant of virus-infected cell cultures using a Dzup Genomic DNA Isolation Reagent (Sangon Biotech, Shanghai, China) following the manufacturer’s protocol. The extracted DNA was sequenced on the Illumina X plus platform (Illumina, USA) using the PE150 strategy by Novogene (Beijing, China). The sequencing data are presented in Supplementary data Fig. S1; Table S2, which confirms that the viruses generated by homologous recombination are in good homogeneity.

### Growth kinetic analysis

To draw the growth curves of different recombinant baculoviruses, *Sf*9 cells were infected with the recombinant viruses expressing GFP at a multiplicity of infection (MOI) of 0.5. The supernatants of the infected cell cultures were harvested at 24, 48, 72, 96, 120, and 144 hours post-infection (hpi). Titers of BV were determined by a TCID_50_ endpoint dilution assay in triplicates, and the growth curves were made using GraphPad Prism software.

### Protein expression and detection

To monitor the expression of GFP in baculovirus-infected cells, GFP fluorescence was observed using an inverted fluorescence microscope (Leica DMi8). *Sf*9 cells were infected with recombinant baculoviruses at an MOI of 3 and harvested for protein detection at 2–4 days post-infection (dpi), or infected at an MOI of 10 and harvested at 12 and 24 hours post-infection (hpi). Protein samples were separated by 10% or 12% sodium dodecyl sulfate-polyacrylamide gel electrophoresis (SDS-PAGE) and stained with Coomassie brilliant blue R-250.

To examine the phosphorylated LEF-6, purified LEF-6 proteins were separated by SuperSep Phos-tag (Wako Pure Chemical Industries) with 12.5% polyacrylamide gels containing 50 µm Phos-tag acrylamide and ZnCl_2_. After electrophoresis, Phos-tag acrylamide gels were washed three times with 1 × transfer buffer (25 mM Tris, 192 mM glycine, and 20% ethanol) containing 10 mM EDTA with gentle shaking for 10 min, and then washed with 1 × transfer buffer without EDTA for 10 min. Proteins were transferred onto PVDF (Millipore) membranes using a semi-dry blotting apparatus.

To detect the recombinant proteins by Western blot, anti-LEF6 rabbit polyclonal antibody (AtaGenix Laboratories Co., Ltd., Wuhan), anti-GFP mouse monoclonal antibody (TransGen Biotech), anti-GAPDH mouse monoclonal antibody (TransGen Biotech), anti-Histone H3 mouse monoclonal antibody (TransGen Biotech), and anti-gp64 (AcV5) mouse monoclonal antibody (Santa Cruz Biotechnology) were used as primary antibodies. HRP-conjugated goat anti-mouse antibody and goat anti-rabbit antibody (CoWin Biotech) were used as secondary antibodies. All antibodies were diluted according to the manufacturer’s instruction. To exclude differences in sample loading amounts, GAPDH was used as an internal control.

### LEF-6 protein purification

To express LEF-6 and its mutants, *lef-6* gene fragment was amplified with primers pTriEx-NcoI-F and pTriEx-XhoI-R and inserted into pTriEx1.1 vector (Novagen) between the *Nco*I and *Xho*I sites, so that the His-tagged protein can be expressed both in *E. coli* cells driven by T7 RNA polymerase promoter and in insect cells driven by baculovirus late gene *p10* promoter. To generate *lef-6* point and truncated mutants, the mutations were introduced by overlapping PCR into the pTriEx/lef6 construct.

To express LEF-6 and its mutants in prokaryotic cells, the recombinant plasmids were respectively transformed into *E. coli* BL21 (DE3) competent cells, which were then induced with isopropyl-β-thiogalactopyranoside (IPTG) at 18°C for protein expression.

To express the protein in insect cells, recombinant baculoviruses were generated by co-transfection of *Sf*9 cells with the pTriEx vectors and linearized Bacmid bAcΔlef6. High Five cells were then infected with the recombinant baculoviruses at an MOI of 5 for 3 days and harvested by centrifugation at 500 × *g* for 5 min. The LEF-6 protein expression was examined by Western blot using anti-LEF6 rabbit polyclonal antibody (AtaGenix Laboratories Co., Ltd., Wuhan).

The His-tagged LEF-6 and its mutants were purified using Ni-Agarose Resin (Cowin Biotech) following the manufacturer’s protocol. A protein phosphatase inhibitor (Solarbio) was added during protein purification to prevent protein degradation. His-tagged GFP was purified from High Five cells infected with vAc expressing GFP driven by *p10* promoter (Fig. 1A), and the protein was used as a control to detect protein-bound nucleic acids.

### Detection of nucleic acids bound with LEF-6 and transcriptome sequencing

To assess the nucleic acid binding property of LEF-6 protein, about 10 µg of LEF-6 protein purified from infected High Five cells was extracted with phenol/chloroform/isoamyl alcohol (25:24:1), and the aqueous fraction was detected by 1% agarose gel electrophoresis and ethidium bromide staining. RNA and DNA bands were determined by digestion with DNase I and RNase A prior to gel analysis.

For transcriptome sequencing, total RNA was extracted from purified proteins using RNA Isolater Total RNA Extraction Reagent (Vazyme Biotech). Library construction, quality control, and high-throughput sequencing were performed by LC-Bio Technologies. The raw RNA-seq reads were filtered and mapped to the AcMNPV (GenBank: MW496365.1) and High Five cell (GenBank: GCA_003590095.1) genome.

### Prediction of LEF-6 structure and its interaction with RNAs

AlphaFold2 Protein Structure Database (http://www.alphafold.ebi.ac.uk) was used to predict the three-dimensional structure of nonphosphorylated LEF-6 protein (25, 26). The reliability of predictive models was evaluated through Local Distance Difference Test (LDDT) score and Predicted Aligned Error (PAE). The LDDT indicator is a per-residue confidence score. Protein regions showing values of LDDT higher than 70 are expected to be modeled with reasonable accuracy. The best-predicted model was visualized and annotated with UCSF Chimera software. Three-dimensional structure and annotation data of TAP (Uniprot No. Q9UBU9) were obtained through the Universal Protein Resource (27) and were visualized using UCSF Chimera software. Structure alignment and superposition of the LEF-6 and TAP were performed using the Foldseek program (https://search.foldseek.com) (28).

To predict the structures for RNA-LEF6 complexes, the amino acid sequences of non-phosphorylated LEF-6 protein, phosphorylated LEF-6, and LEF6_S95A_ were submitted to the server (https://golgi.sandbox.google.com) for protein structural prediction by AlphaFold3 (29), and were then docked with *p6.9* ssRNA, *p10* ssRNA, and *ie1* ssRNA, respectively. These predicted models were downloaded in CIF format and visualized using PyMOL software.

### Electrophoretic mobility shift assay (EMSA)

The mixture of 20 µM RNA and 0, 2, 4, or 6 µg of protein diluted in EMSA binding buffer (10 mM Tris pH 7.5, 20 mM KCl, 1 mM MgCl_2_, 1 mM DTT) was incubated for 30 min at 22°C. The RNA-protein complexes were then separated by electrophoresis on 1% agarose gel in Tris acetate-EDTA (TAE) buffer for 15 min. The gel was stained with ethidium bromide and visualized under ultraviolet light.

### Fluorescence anisotropy analysis

FAM(6-carboxyfluorescein)-labeled RNAs, 5′-GACAGGUCGCCGAAGUUACAGAACACG-3′ (*p6.9* mRNA fragment), 5′-CGGUUUGCCCGCUCAAUUGACCGAUCU-3′ (*p10* mRNA fragment), and 5′-UUAUAACCAUCCCACCCCGGAUGGAGC-3′ (*ie1* mRNA fragment) were synthesized by Sangon Biotech. The *p6.9* and *p10* ssRNAs are chosen from the two peaks of mRNAs, which were co-purified with LEF-6 protein and had the highest reads in the peaks in RNAseq. The *ie1* ssRNA is chosen from the early gene *ie1* mRNA with the same GC content as the *p6.9* ssRNA and *p10* ssRNA. An unrelated ssRNA fragment (5′-UUUUUUUUUUCUCUGCUCGACG-3′) was gifted by professor Ximiao Hou and was used as a control. The binding buffer (20 mM Tris-HCl, pH 7.5, 20 mM NaCl) containing FAM-labeled ssRNA (5 nm) was added to the wells of a black 96-well plate. Serially diluted protein was added to the wells to a final reaction volume of 150 µL. Each sample was allowed to equilibrate in solution at 28°C for 5 min prior to measurement using an Infinite F200 instrument (TECAN) at the excitation wavelength of 494 nm and emission wavelength of 518 nm. Fluorescence anisotropy was calculated using Magellan7 software.

### Nuclear-cytoplasmic fractionation and quantitative real-time PCR (qPCR)

Infected cells were fractionated into cytoplasmic and nuclear fractions using the NE-PER Nuclear and Cytoplasmic Extraction Reagents kit (Thermo Fisher Scientific) according to the manufacturer’s protocol. Total RNA was then extracted from the fractions using RNA Isolater Total RNA Extraction Reagent (Vazyme Biotech). The purified RNA was subjected to reverse transcription (RT) reactions with HiScript III 1st Strand cDNA synthesis kit (Vazyme Biotech). The cDNA was used for real-time PCR using 2 × Q3 SYBR qPCR Master Mix (Tolo Biotech). The primer sequences used for RT-qPCR are listed in Table S1. Two biological replicates were measured in duplicate for each experimental group, with a minimum of two independent experiments. The percentage ratio of target mRNA in the nucleus and cytoplasm was calculated according to the 2^−∆∆Ct^ method.

### Subcellular localization of LEF-6

Based on pBac5 plasmid (Novogen), a fragment containing the *OpIE2* promoter (amplified from pIZ-V5-His plasmid) and *gfp* gene was inserted into pBac5 between *Bgl*II and *Sph*I sites, and the construct was named as pBac5/OpIE2p-GFP. For transient expression of LEF6-GFP fusion protein, the lef6 fragment or lef6_100-104A_ fragment was inserted into the vector between the *OpIE2* promoter and *gfp* gene. These constructed plasmids were named pBac5/OpIE2p-lef6-GFP and pBac5/OpIE2p-lef6_100-104A_-GFP. To detect the subcellular localization of LEF6-GFP in the absence of virus infection, *Sf*9 cells transfected with pBac5/OpIE2p-lef6-GFP or pBac5/OpIE2p-lef6_100-104A_-GFP were stained with Hoechst 33342 (Beyotime Biotechnology). Images were taken by confocal fluorescence microscope (Nikon AX). The distribution of LEF-6 proteins in AcMNPV-infected cells was examined by nuclear-cytoplasmic fractionation and Western blot.

### Phosphorylation analysis by mass spectrometry

Purified LEF-6 protein (20 µL) was separated by electrophoresis on a 10% SDS-PAGE gel, and protein bands were visualized by Coomassie brilliant blue R-250 staining. The protein bands were cut from the gel and washed with ultrapure water. For in-gel tryptic digestion, the protein/gel mixture was washed with a mixture of NH_4_HCO_3_ (50 mM, pH 8.0) and 50% acetonitrile until clear. Gel pieces were dehydrated with 100 µL of 100% acetonitrile for 5 min, and the liquid was removed. Protein reductive alkylation and enzymatic hydrolysis were performed by the subsequent addition of dithiothreitol (DTT, 10 mmol/L), iodoacetamide (IAM, 55 mmol/L), and 10 ng/µL trypsin.

The tryptic peptides were dissolved in 0.1% formic acid and directly loaded onto a home-made reversed-phase analytical column. The incremental gradient was comprised of formic acid in acetonitrile, at a constant flow rate of 400 nL/min on an EASY-nLC 1000 UPLC system. The peptides were analyzed using an NSI source followed by tandem mass spectrometry (MS/MS) in Q Exactive Plus (Thermo) coupled online to the UPLC.

The MS/MS data were processed using Proteome Discoverer 2.4. Tandem mass spectra were searched against LEF-6 protein sequence. Phosphorylated peptides were identified based on the mass-to-charge ratio (m/z) of the fragment ion on the peptide spectrum.

## RESULTS

### LEF-6 is required for the efficient replication of AcMNPV

To investigate the role of LEF-6 in AcMNPV infection, the gene was deleted from bacmid bAc. The resultant bacmid bAcΔlef6 was linearized and co-transfected with pBac5/p10p-GFP (KO), pBac5/p10p-GFP/lef6p-lef6 (REP), and pBac5/p10p-GFP/gp64p-lef6p-lef6 (OE) into *Sf*9 cells to generate recombinant baculoviruses vAcΔlef6 (KO), vAcΔlef6/lef6p-lef6 (REP), and vAcΔlef6/gp64p-lef6p-lef6 (OE). This approach allows the effects of *lef-6* deletion, complementation, and overexpression on viral late gene expression in the virus-infected cells to be easily examined through GFP reporter expression under the control of the viral very late gene *p10* promoter. The schematic diagram of the viral and plasmid vectors is shown in Fig. 1A. Linearized bAc bacmid was co-transfected with pBac5/p10p-GFP to produce a control virus vAc (WT).

The *lef-6* gene has been reported as nonessential for viral replication, but it can accelerate late gene transcription in *Sf*9 cells (10). Consistent with the previous study, the growth curves determined in our study showed that the yield of vAcΔlef6 was about 10-fold lower than vAc (Fig. 1B), and the deletion of *lef-6* did not affect the viral DNA replication (Fig. S2). The virus replication ability of vAcΔlef6/lef6p-lef6 was restored by complementation of *lef-6* under the control of its native promoter at the *ph* locus in the viral genome. The virus overexpressing *lef-6* (vAcΔlef6/gp64p-lef6p-lef6) had a similar growth curve to vAc and vAcΔlef6/lef6p-lef6 (Fig. 1B). These findings suggest that the expression of the *lef-6* gene is beneficial for the efficient multiplication of AcMNPV in cultured cells.

### LEF-6 promotes late gene expression

To determine whether the *lef-6* knockout affects the expression of other late genes, we also generated *gfp* expression vectors and recombinant viruses using *p6.9* promoter as a representative of late gene to replace the very late *p10* promoter (Fig. 1A). *Sf*9 cells were infected with vAcΔlef6 and vAc in parallel at an MOI of 3, and the expression of GFP was visualized by using a fluorescence microscope at 2–4 dpi. To examine the GFP expression at earlier times of 12 and 24 hpi, the cells were infected with the recombinant viruses at 10 MOI. The results showed that the production of GFP in the vAc-infected cells was not detectable at 12 hpi but was detectable at 24 hpi under the fluorescence microscope (Fig. S3). In contrast, the GFP fluorescence was hardly visible until 48 hpi in vAcΔlef6-infected cells, suggesting that the deletion of the *lef-6* gene severely inhibited the expression of GFP that was driven by late (*p6.9*) (Fig. S3A) and very late (*p10*) promoters (Fig. S3B). Western blot analyses confirmed the diminished levels of GFP protein expression under the regulation of the *p6.9* promoter (Fig. 1C) or *p10* promoter (Fig. 1D) in vAcΔlef6-infected cells.

To restore the *lef-6* gene, the ORF of *lef-6* along with a 105 bp fragment upstream—identified as its native promoter (2)—was inserted into the *lef6*-KO viral genome at the *ph* locus in the viral genome (Fig. 1A). The replication ability of the REP virus was restored by complementation of *lef-6* (Fig. 1B). However, unexpectedly, we detected that *lef-6* was expressed at much lower levels in the REP than the WT. Even so, the GFP expression driven by both the late and very late promoters of REP viruses was restored to levels comparable to the WT virus (Fig. 1C and D), suggesting that even a low level of LEF-6 was sufficient to promote the expression of viral late proteins. The tandem *gp64-lef6* promoter fully restored the expression of *lef-6*, and the GFP production was slightly elevated under the control of late *p6.9* promoter (Fig. 1C) but dramatically accelerated by the very late *p10* promoter (Fig. 1D).

In natural AcMNPV infection, the most abundant very late transcripts are *polyhedrin* (*ph*), which is nonessential for the virus infection in cultured cells; therefore, the gene is normally deleted, but the strong *ph* promoter is often used for recombinant protein expression in BEVS. To investigate whether the expression of LEF-6 affects the gene expression controlled by the *ph* promoter, a set of WT, KO, REP, and OE viruses carrying the *ph* promoter-driven *gfp* expression cassette was generated (Fig. S4A). The results showed that LEF-6 was also involved in the upregulation of gene expression controlled by *ph* promoter (Fig. S4B and C). These data support the function of LEF-6 as a regulation factor for the expression of viral late and very late genes.

### LEF-6 protein is a viral RNA-binding protein

**Fig 2:**
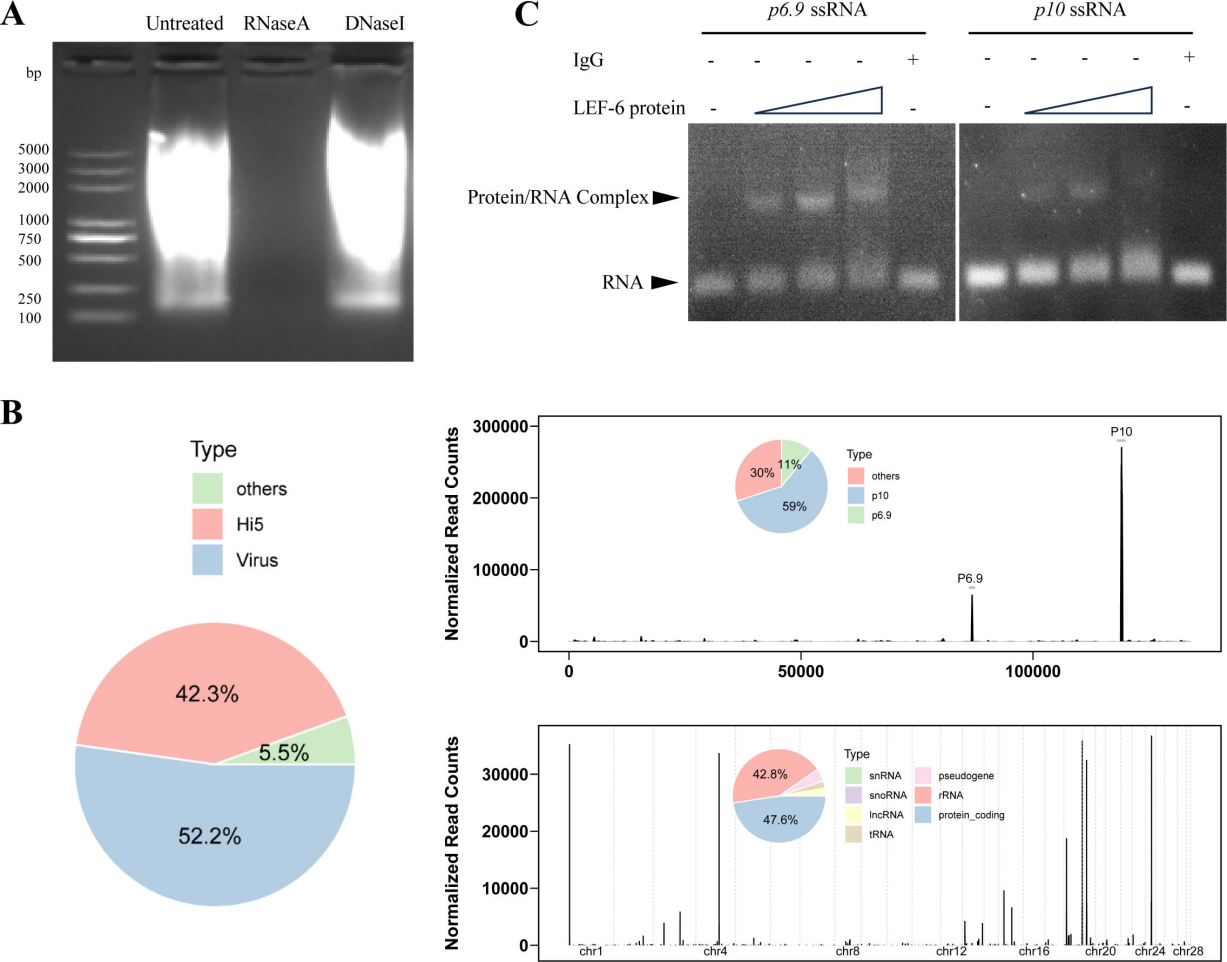
Determination of RNA molecules bound with LEF-6. (**A**) Characterization of the nucleic acid type bound with purified LEF-6 protein. The LEF-6 protein was purified with Ni-Agarose Resin from baculovirus-infected High Five cells. Protein-bound nucleic acids were extracted using phenol/chloroform/isoamyl alcohol (25:24:1), digested with RNase A or DNase I, and analyzed by agarose gel electrophoresis and ethidium bromide staining. (**B**) Analysis of the RNAs bound with LEF-6 by RNA-seq. The distribution of the sequencing reads along the AcMNPV (upper panel) and Tn cell (lower panel) genomes is shown. The gene types detected are shown in the inserted charts. (**C**) Verification of the binding ability of LEF-6 to *p6.9* and *p10* mRNA fragments by EMSA.

To investigate whether LEF-6 is associated with nucleic acids, we purified His-tagged LEF-6 from AcMNPV-infected High Five cells at 3 dpi using Ni-Agarose Resin. The nucleic acids co-purified with the LEF-6 protein were extracted with phenol/chloroform/isoamyl alcohol (25:24:1), and the aqueous fraction was detected by agarose gel electrophoresis and ethidium bromide staining. Smeared nucleic acid bands were observed in the sample extracted from purified LEF-6 (Fig. S5). After respective treatment with RNase A and DNase I, the nucleic acids co-purified with LEF-6 protein were degraded with RNase A but tolerated the digestion of DNase I (Fig. 2A), suggesting that LEF-6 protein was an RNA-binding protein.

To identify the RNA fragments associated with LEF-6, the RNAs co-purified with LEF-6 protein were analyzed by deep sequencing. It was revealed that the majority of the sequencing reads (52.2% of the total reads) were mapped to the baculovirus genome. Among them, two predominant peaks, the *p10* and *p6.9* mRNAs, accounted for 59% and 11% of the total viral RNA reads (Fig. 2B). Although some cellular RNAs (mostly rRNAs and mRNAs) were also co-purified with LEF-6, they were scattered along the cell genome, and the highest peak was much lower than *p10* and *p6.9* mRNAs. In AcMNPV-infected Tnms42 (a subclone of High Five) cells, *odv-e18* has been found to have a close transcription level to *p6.9* in late infection (2). By quantitative RT-PCR, it was shown that the relative expression level of *p6.9* mRNA was about 1-fold higher than that of *odv-e18* mRNA in AcMNPV-infected High Five cells at 3 dpi. In comparison, *odv-e18* mRNA was hardly detectable in the RNA sequencing data of RNAs co-purified with LEF-6 (Fig. S6). The RNA sequencing data suggest that LEF-6 may preferentially bind to some viral RNAs, especially *p10* and *p6.9* mRNAs.

In order to explore whether LEF-6 protein binds to *p6.9* and *p10* RNAs *in vitro*, ssRNAs of 27 nt in length from the two peaks detected by RNAseq were synthesized, and EMSA assays were performed. The results showed that the migration of both *p6.9* ssRNA and *p10* ssRNA was obviously retarded in the presence of LEF-6 protein (Fig. 2C), confirming that LEF-6 could interact with these RNA fragments from viral late and very late genes.

### The LEF-6 RNA-binding region locates in the N-terminal half, which is not enough to maintain its function

**Fig 3:**
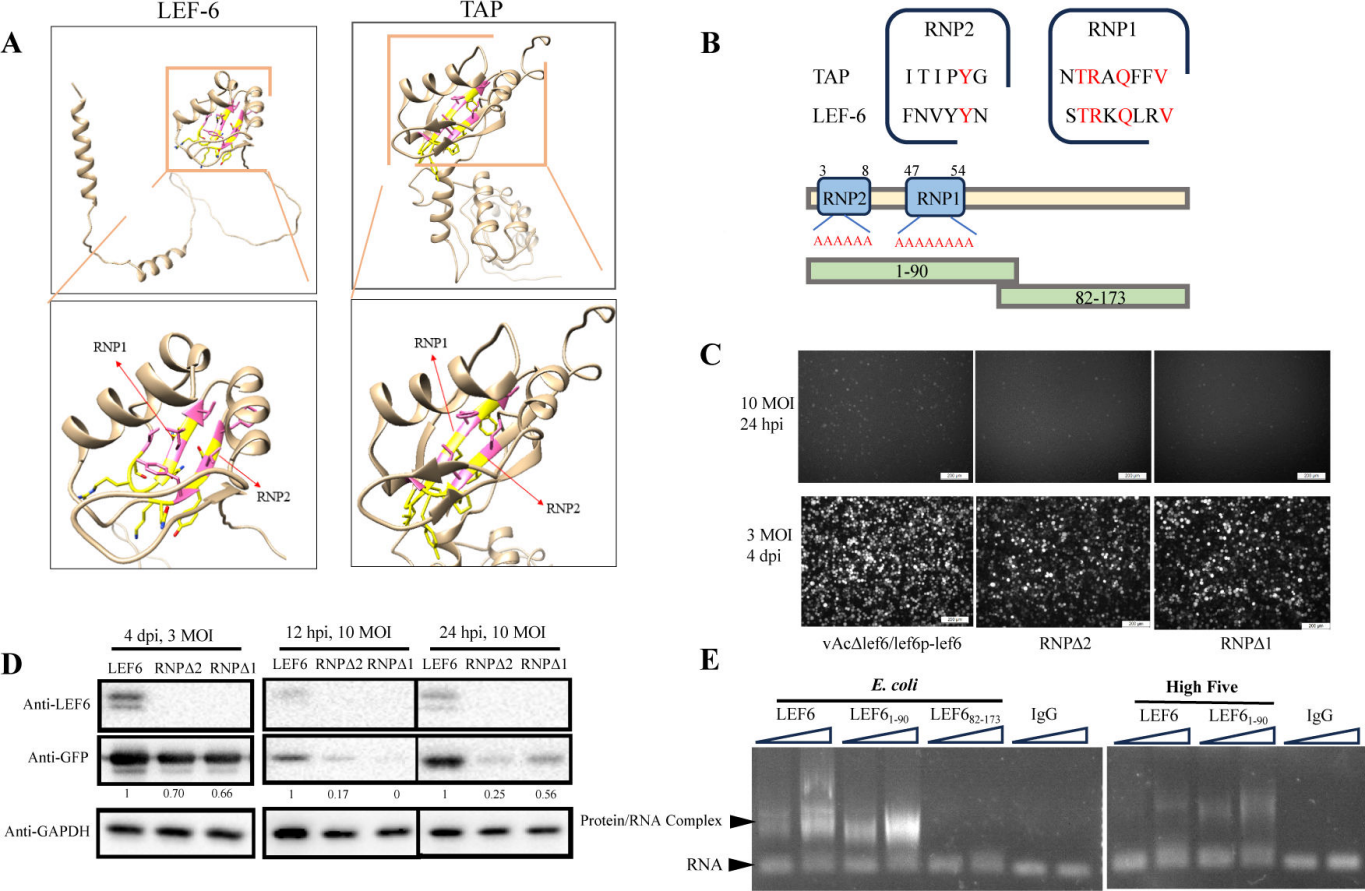
Identification of the N-terminal region as the RNA-binding domain of LEF-6. (**A**) Comparison of the structure of LEF-6 and TAP (Uniprot accession number Q9UBU9). Structure of LEF-6 was predicted using AlphaFold2. The arrows indicate the RNP1 and RNP2 regions. UCSF Chimera software was used to display the side chain structure of amino acid. The amino acids with side chains pointing outward are shown in yellow, and the amino acids with side chains pointing inward are shown in pink. (**B**) Diagram of the LEF-6 RNP mutants and truncated proteins generated for the identification of RNA-binding domain. The predicted RNP1 and RNP2 motifs of LEF-6 were identified by sequence alignment to TAP. RNPΔ1 and RNPΔ2 mutants were constructed by substitution of the residues in the RNPs into alanines. LEF6_1-90_ and LEF6_82-173_ are produced as the N-terminal half and C-terminal half truncated proteins. (**C**) Observation of the GFP reporter expression under the control of the *p10* promoter by fluorescence microscopy. *Sf*9 cells were infected with the indicated viruses at an MOI of 3 and examined at 4 dpi, or at an MOI of 10 and examined at 24 hpi.(**D**) Examination of the protein levels in cells infected by the viruses expressing the indicated LEF-6 mutants. Total cell lysates were separated by SDS-PAGE and respectively probed by anti-LEF6, anti-GFP and anti-GAPDH antibody. GAPDH was detected as a loading control. The GFP bands were quantified by densitometry scanning using Image J software, and the relative expression levels to the WT are shown below the GFP bands. (**E**) Determination of the RNA-binding property of the truncated LEF-6 by EMSA. Synthesized *p6.9* ssRNA was used as the RNA ligand. LEF6 and LEF6_1-90_ were purified from *E. coli* or High Five cells. LEF6_82-173_ was purified from *E. coli*. IgG was used as a control protein.

It has been reported that the encoding region of LEF-6 has more than 80% probability of being related to the retroviral mRNA export factor TAP, although TAP is a bigger protein than LEF-6 (3, 30). In order to identify the RNA-binding domain of LEF-6 protein, we used AlphaFold2 (25, 26) to predict the protein structure of LEF-6 (Fig. 3A). Using Foldseek (28), it was revealed that the N-terminal half of LEF-6 (amino acids 2–72) folds into a domain with a very similar structure with the RNA-binding domain of TAP (Fig. 3A; Fig. S7). The N-terminal half of LEF-6 folds into three-stranded antiparallel β-sheets with a right-handed twist, and two perpendicularly oriented α-helices pack against one side of the β-sheets, whereas the other side of the β-sheets is exposed to solvent. The βαββαβ topology of this N-terminal domain is similar to the structure of the TAP RNA-binding domains containing two ribonucleoprotein (RNP) motifs. By aligning the primary sequences of RNP1 and RNP2 between LEF-6 and TAP, we identified some conserved amino acids in the candidate RNP1 and RNP2 motifs of LEF-6 (Fig. 3B). By substituting the residues in the RNPs into alanines, two recombinant baculoviruses, each lacking one RNP region (RNPΔ1 and RNPΔ2), were generated. Compared to the parental baculovirus, GFP expression driven by the *p10* promoter declined in the RNP mutants (Fig. 3C and D). Meanwhile, we observed that the LEF-6 protein dropped to undetectable levels by Western blot (Fig. 3D), indicating that the mutation of the RNPs disrupted the stability of LEF-6 and therefore impeded its function in enhancing viral late gene expression.

To verify the postulated RNA-binding property of the LEF-6 N-terminal domain, we generated two truncated proteins, each containing the N-terminal and C-terminal half of LEF-6. LEF6_1-90_, encompassing the predicted RNA-binding domain with βαββαβ structures and two RNP motifs (Fig. 3A), was successfully expressed and purified from both *E. coli* and High Five cells (Fig. S8). For the C-terminal LEF6_82-173_, which is predicted to contain two helices and some random coils, we got the purified protein from *E. coli* (Fig. S7) but failed to purify the protein from the insect cells (data not shown). The expected molecular weights of LEF6_82-173_ and LEF6_1-90_ proteins are both 12.1 kDa. However, in SDS-PAGE gels, we observed that the migration rate of LEF6_82-173_ protein purified from *E. coli* cells was much slower than that of LEF6_1-90_ (Fig. S8). The proportion of acidic amino acids in LEF6_1-90_ is 8%, while the ratio in LEF6_82-173_ is 21%. It has been reported that the larger the proportion of acidic amino acids, the higher the apparent molecular weight in SDS-PAGE (31). Therefore, the high number of acidic amino acids in LEF6_82-173_ may contribute to its relatively slower-than-expected migration of the protein. By EMSA, the results demonstrated that LEF6_1-90_ from both *E. coli* and High Five cells bound to *p6.9* ssRNA *in vitro*, while LEF6_82-173_ failed to retard the migration of the RNA band (Fig. 3E), confirming the location of the RNA-binding domain at the N-terminal half of LEF-6.

**Fig 4:**
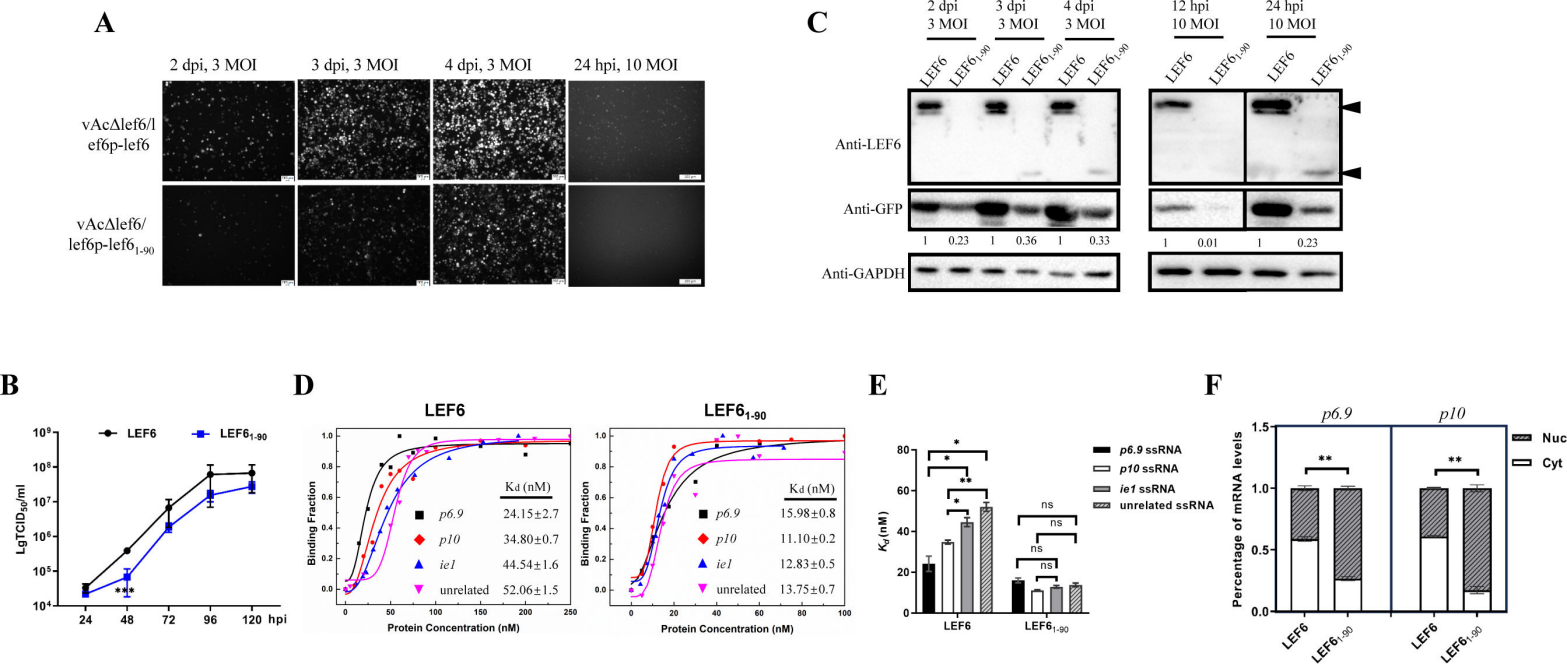
The effects of LEF-6 C-terminal truncation on baculovirus infection, RNA binding, and mRNA trafficking. (**A**) Observation of virus infection and late gene expression by fluorescence microscopy. *Sf*9 cells were infected with the indicated recombinant viruses at an MOI of 3 and observed at 2, 3, and 4 dpi, or infected at 10 MOI and observed at 24 hpi. (**B**) Growth curves of vAcΔlef6/lef6p-lef6_1-90_ vs. vAcΔlef6/lef6p-lef6. *Sf*9 cells were infected at an MOI of 0.5. The cell culture supernatants were harvested at intervals of 24 hours, and the viral titers were determined by an endpoint dilution assay in triplicate. (**C**) Protein expression levels determined by Western blot. Total cell lysates were separated by SDS-PAGE and probed using anti-LEF6, anti-GFP and anti-GAPDH antibody. GAPDH was detected as a loading control. The GFP bands were quantified by densitometry scanning using Image J software, and the relative expression levels to the WT are shown below the GFP bands. (**D and E**) Determination of the RNA-binding property of LEF-6 and LEF6_1-90_ to ssRNAs by fluorescence anisotropy measurements. The binding curve and *K_d_* values calculation were performed by using Magellan 7 software. These data are shown as the means ± SD of two replicates. (**F**) Examination of the viral mRNA distribution between the cytoplasm and nucleus. *Sf*9 cells were infected with vAcΔlef6/lef6p-lef6 or vAcΔlef6/lef6p-lef6_1-90_ at an MOI of 3. Nuclear-cytoplasmic fractionation was performed at 36 hpi. The mRNA levels were quantified by RT-qPCR. Data were presented as the means ± SD of two replicates. ns: not significant, *P  <  0.05, **P  <  0.01, ***P < 0.001.

Subsequently, a recombinant virus vAcΔlef6/lef6p-lef6_1-90_ was generated by replacing the full-length *lef-6* gene in vAcΔlef6*/*lef6p-lef6 with the truncated gene expressing only the N-terminal half domain of LEF-6. The infection of *Sf*9 cells revealed that deletion of the amino acids 91–173 of LEF-6 did not severely affect virus production, but the fluorescence intensity of GFP representing the expression of viral late gene was significantly reduced (Fig. 4A). From the viral growth curves, it showed that the titers of vAcΔlef6/lef6p-lef6_1-90_ were consistently lower than its parental virus, although a significant difference was only detected at 2 dpi (Fig. 4B). Western blot analyses confirmed that the expression of GFP protein, under the regulation of the *p10* promoter, was significantly impaired in the *Sf*9 cells infected with vAcΔlef6/lef6p-lef6_1-90_ (Fig. 4C). Based on these data, it appears that the C-terminal half, in addition to the N-terminal half, is required for LEF-6 to fulfill its function in late gene regulation, even though the RNA-binding domain is located in the N-terminal half of the protein.

Fluorescence anisotropy measurements showed that the LEF-6 protein exhibited significantly higher binding affinity to late gene *p6.9* ssRNA and very late gene *p10* ssRNA, compared with the early gene *ie1* ssRNA and an unrelated ssRNA. In contrast, the truncated LEF6_1-90_ protein exhibited similar and robust binding ability to all the four ssRNAs (Fig. 4D and E).

Since TAP is a mRNA export factor which can transport retroviral mRNA out of cell nucleus, we postulate that LEF-6 may play a similar role in baculovirus infection, and its viral RNA trafficking ability may be impaired for the truncated LEF6_1-90_ that cannot preferentially bind to viral mRNAs. To explore the mRNA trafficking property of LEF-6, we examined the distribution of *p6.9* mRNA and *P10* mRNA between the cytoplasm and nucleus of *Sf*9 cells, which were infected by vAcΔlef6/lef6p-lef6 and vAcΔlef6/lef6p-lef6_1-90_, respectively, at 36 hpi. After nuclear-cytoplasmic fractionation, the mRNA levels were quantified by RT-qPCR. The results showed that about 60% of the viral mRNAs were transported into the cytoplasm with the presence of full-length LEF-6. When LEF6_1-90_ was expressed, more than 70% of the mRNAs were retained in the nucleus (Fig. 4F). These data confirm that LEF-6 is involved in viral mRNA trafficking, but the RNA-binding domain is not enough to fulfill its protein function.

### Identification of the nuclear localization sequence (NLS) in LEF-6

**Fig 5:**
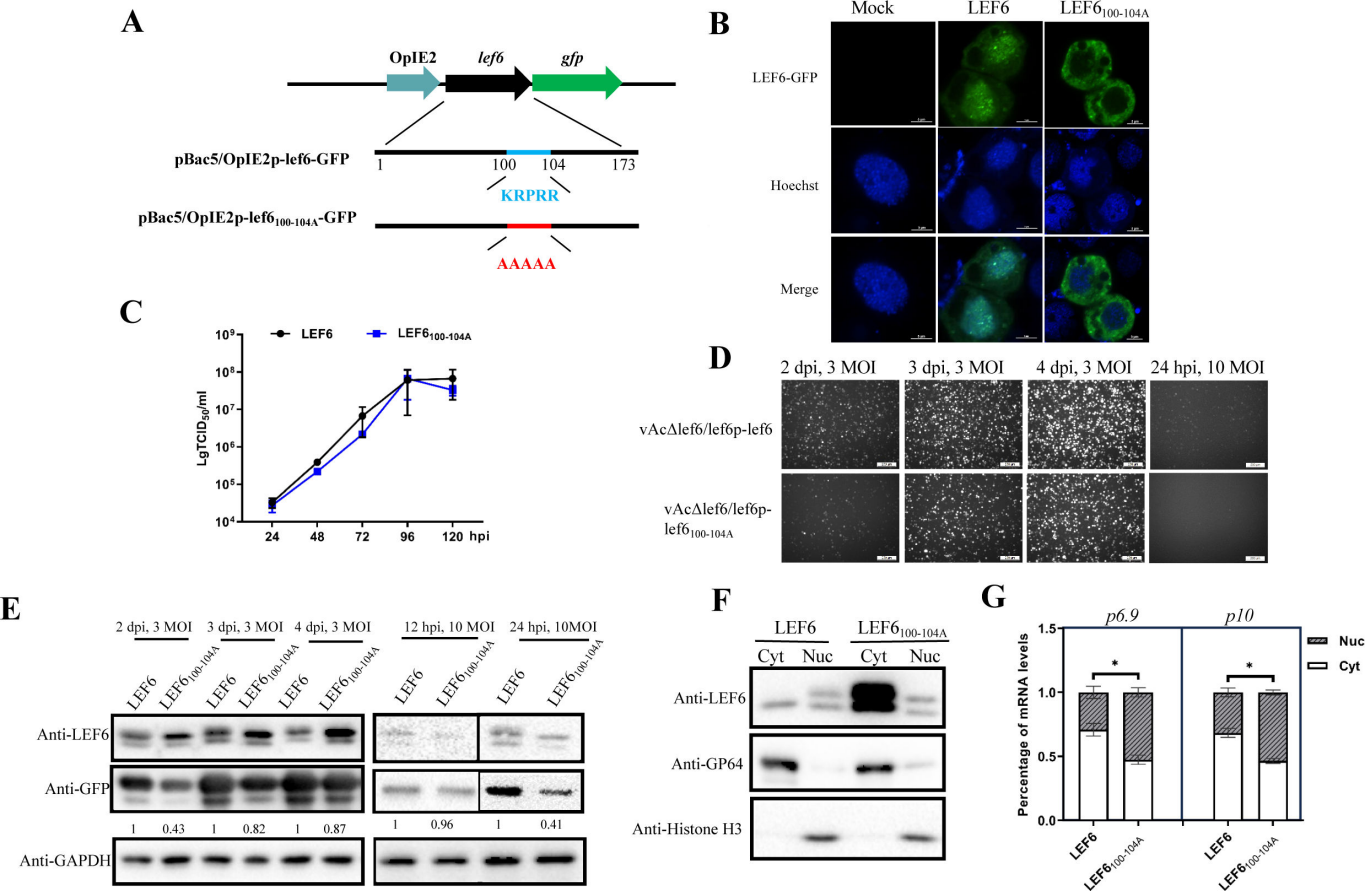
Identification of the LEF-6 NLS motif and its role in virus replication. (**A**) Diagram of the plasmids constructed for transient expression of LEF-6 and GFP fusion proteins. The fused gene fragment was expressed under the control of the *OpIE2* promoter. (**B**) Observation of the subcellular localization of GFP-tagged LEF-6 and LEF6_100-104A_ in transfected *Sf*9 cells. Nuclei were stained with Hoechst dye. Bar, 5  µm. (**C**) Growth curves of vAcΔlef6/lef6p-lef6_100-104A_ vs. vAcΔlef6/lef6p-lef6. *Sf*9 cells were infected by the virus at an MOI of 0.5. The cell culture supernatants from the infected cells were harvested at intervals of 24 hours, and the viral titers were determined by an endpoint dilution assay in triplicate. (**D**) Observation of GFP expression by fluorescence microscopy. *Sf*9 cells were infected with the indicated viruses at an MOI of 3 and observed at 2, 3, and 4 dpi, or infected at 10 MOI and observed at 24 hpi. (**E**) Detection of protein expression levels by Western blot. The total cell lysates were respectively probed with anti-LEF6, anti-GFP, and anti-GAPDH antibodies. GAPDH was used as a loading control. (**F**) Detection of the protein levels in the fractionated cytoplasmic and nuclear samples by Western blot. GP64 was detected as a reference protein in the cytoplasmic fraction and Histone H3 as a nuclear protein. (**G**) Examination of the viral mRNA distribution between the cytoplasm and nucleus by RT-qPCR. Data were presented as the means ± SD of two replicates. *P  <  0.05.

Previous studies have shown that the LEF-6 protein is localized mainly in the nucleus of infected cells, with a portion located in the cytoplasm (10). By amino acid sequence analysis, amino acids 100–104 containing four basic amino acids (KRPRR) were identified as a potential NLS motif. To confirm the subcellular localization of LEF-6 and its NLS motif, LEF6-GFP and LEF6_100-104A_-GFP fusion proteins were transiently expressed by transfecting the corresponding plasmids into *Sf*9 cells (Fig. 5A). From the images taken under a confocal microscope, it showed that GFP-fused LEF-6 was distributed mainly in the nucleus, consistent with the subcellular localization of LEF-6 in infected cells observed by immunofluorescence microscopy (10). In contrast, the GFP-fused LEF6_100-104A_ was mainly localized in the cytoplasm (Fig. 5B). To exclude the possibility that cleavage of the GFP fusion proteins resulted in false fluorescence signals on LEF-6 localization, the transfected cell lysates were examined by Western blot. Full-length LEF6-GFP and LEF6_100-104A_-GFP fusion proteins were detected as the predominant products by both anti-LEF6 and anti-GFP antibodies (Fig. S9). Therefore, the results suggested that the NLS KRPRR efficiently mediated the import of LEF-6 into the nucleus.

To further investigate the role of the KRPRR motif in virus infection, vAcΔlef6/lef6p-lef6_100-104A_, a recombinant virus expressing LEF6_100-104A_ mutant under the control of *lef-6* native promoter and a GFP reporter driven by *p10* promoter, was generated. Time-course analysis of the viral titers showed that mutation of the NLS motif did not significantly affect the virus replication (Fig. 5C). However, the expression levels of late gene reporter GFP were notably lower in the cells infected with vAcΔlef6/lef6p-lef6_100-104A_ than those of the vAcΔlef6/lef6p-lef6 (Fig. 5D and E). In addition, we also observed that the expression levels of LEF6_100-104A_ were much higher than LEF-6 by Western blot even though both were driven by the same promoter (Fig. 5E). Nuclear-cytoplasmic fractionation revealed that a major amount of LEF6_100-104A_ proteins accumulated in the cytoplasm of the cells infected with vAcΔlef6/lef6p-lef6_100-104A_. From the same sample, a portion of LEF6_100-104A_ was able to enter the nucleus, and its protein level in the nucleus was close to LEF-6 (Fig. 5F). A comparable amount of LEF6_100-104A_ and LEF-6 entered the nucleus may explain why the virus production was not obviously impaired by the NLS mutation.

Quantification analysis of the *p6.9* and *p10* mRNAs revealed that lower proportions of *p6.9* and *p10* mRNAs were exported into the cytoplasm of *Sf*9 cells expressing LEF6_100-104A_, compared with the cells producing LEF-6 (Fig. 5G). This result was consistent with the reduced expression of GFP driven by the *p10* promoter in cells infected with vAcΔlef6/lef6p-lef6_100-104A_ (Fig. 5D and E).

### LEF-6 is highly phosphorylated

**Fig 6:**
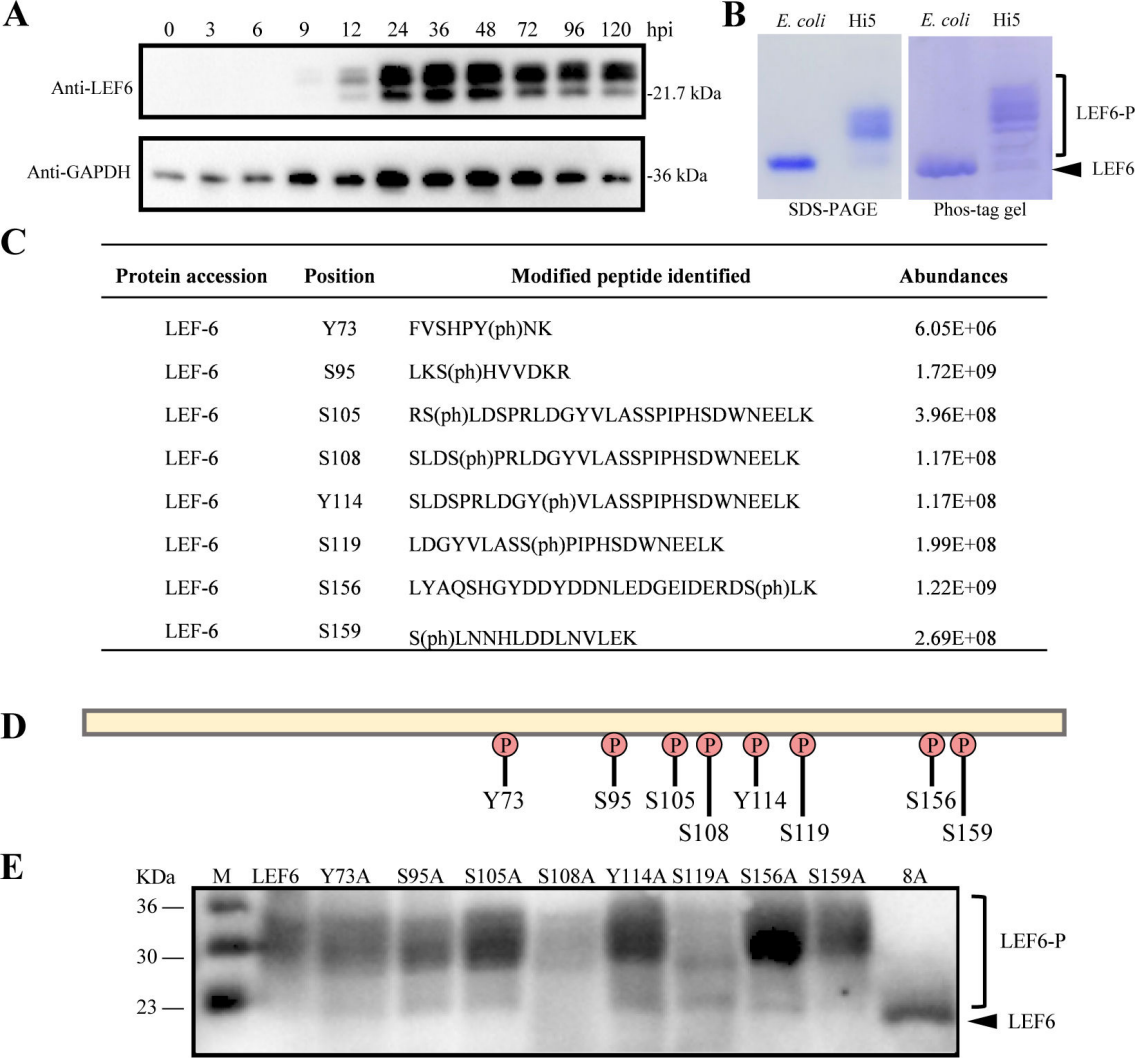
Determination of the phosphorylation sites on LEF-6. (**A**) Time course of LEF-6 expression in baculovirus infection. *Sf*9 cells were infected with vAc at an MOI of 3, and the total cell lysates were probed by anti-LEF6 and anti-GAPDH antibody at the indicated time points. GAPDH was detected as a protein loading control. (**B**) SDS-PAGE and Phos-tag PAGE gel analyses of purified LEF-6. The proteins were purified from *E. coli* or High Five cells. The band consistent with the molecular weight of unmodified target protein is indicated by a black arrow, and the phosphorylated form proteins are indicated by a right square bracket. (**C**) Phosphorylated amino acids identified by MS/MS. LEF-6 protein was purified from baculovirus-infected High Five cells. (**D**) Schematic diagram illustrating the phosphorylation positions on LEF-6. (**E**) Phos-tag gel analysis of the LEF-6 mutants. The indicated mutants were purified from baculovirus-infected High Five cells and detected by Western blot using an antibody against LEF-6.

To investigate the temporal expression pattern of LEF-6 during virus infection, *Sf*9 cells were infected with vAc at an MOI of 3 and harvested at 0, 3, 6, 9, 12, 24, 36, 48, 72, 96, and 120 hours post-infection (hpi). By Western blot, the expression of LEF-6 was detected from 9 hpi, and the protein reached a peak level at about 36 hpi (Fig. 6A). It is noteworthy that irrespective of whether LEF-6 was detected by Western immunoblots (Fig. 1, 3 to 6A) or the purified protein was detected by Coomassie blue staining (Fig. S5A and S8B; Fig. 6B), at least two bands were detected for LEF-6 produced in insect cells. Also, most of the slower-migrating protein bands always migrated at a molecular size higher than expected for the predicted molecular size, perhaps due to differences in phosphorylation. LEF-6 of BmNPV has been reported to be highly phosphorylated in the virus-infected cells (21). The amino acid sequence of AcMNPV LEF-6 is 95% identical to that of BmNPV LEF-6, and it also has multiple potential phosphorylation sites by sequence analysis. To verify the phosphorylation modification of LEF-6, we separated the LEF-6 protein purified from High Five cells using a Phos-tag gel, which detected multiple phosphorylated bands and only a small portion of the protein migrated at the predicted unmodified molecular size (Fig. 6B, right panel), suggesting that AcMNPV LEF-6 was highly phosphorylated.

Phosphoproteomic analysis identified eight possible phosphorylation sites on LEF-6 (Fig. 6C; Fig. S10). Notably, seven of the eight phosphorylated amino acids were located in the C-terminal half of LEF-6 (Fig. 6D). To confirm the phosphorylation sites on LEF-6, we constructed a panel of recombinant baculoviruses overexpressing LEF-6 mutants, each containing a single mutation of the modified amino acid to alanine, as well as an 8A mutant with all the eight phosphorylation sites substituted to alanines. After purification from the virus-infected insect cells and separation on a Phos-tag gel, multiple bands were observed for each of the eight LEF-6 mutants containing single amino acid substitutions. However, only the 8A mutant exhibited a predominant single band with the predicted molecular weight of unmodified LEF-6 (Fig. 6E), confirming the phosphorylation modification sites of AcMNPV LEF-6.

### Identification of the key LEF-6 phosphorylation site involved in the regulation of late gene expression

**Fig 7:**
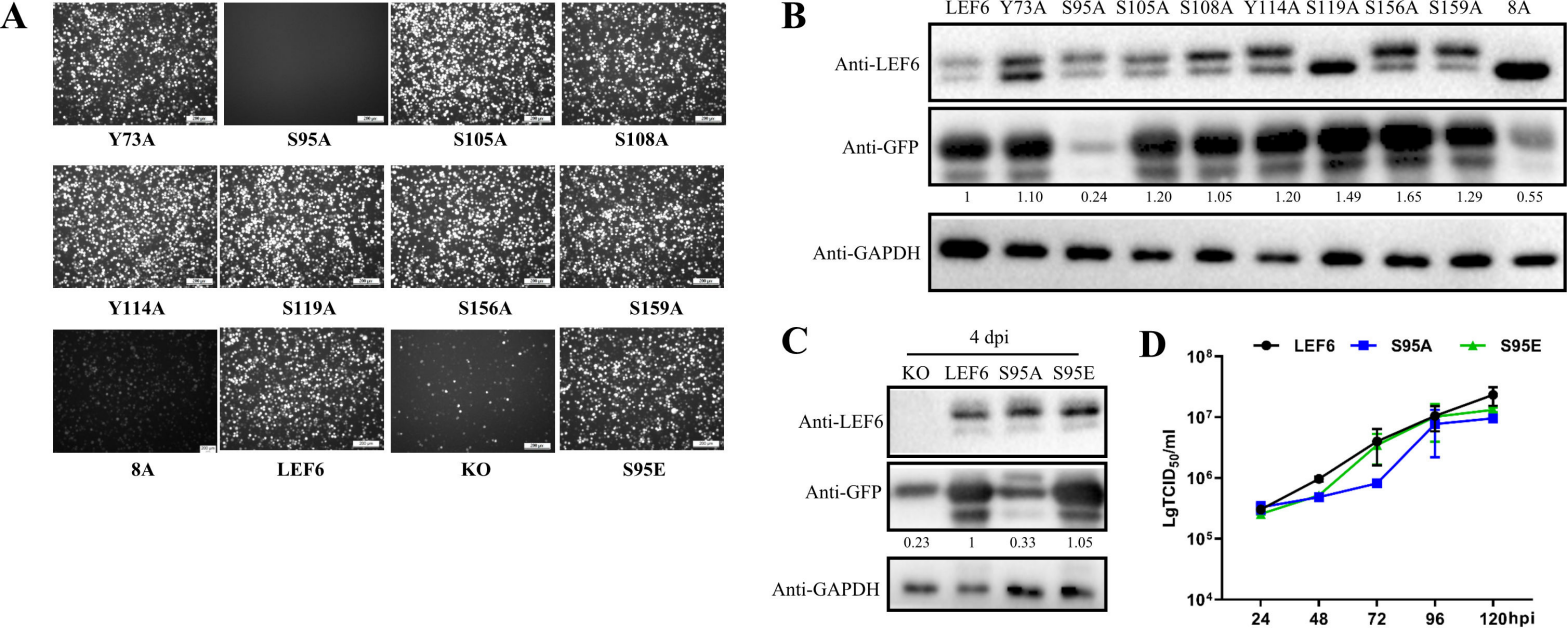
Identification of S95 as a key phosphorylation site involved in late gene expression regulation. (A) Observation of the impact of dephosphorylation and simulated phosphorylation of LEF-6 on the expression of GFP reporter by fluorescence microscopy. *Sf*9 cells were infected with the baculoviruses expressing the indicated LEF-6 mutants at an MOI of 3, and fluorescence images were taken at 4 dpi. (B) Examination of the impact of LEF-6 dephosphorylation on GFP expression by Western blot. (C) Comparison of the influences of LEF-6 dephosphorylation (S95A) and simulated phosphorylation (S95E) on late gene expression by Western blot. (D) Growth curves of the baculoviruses expressing the indicated LEF-6 mutants. *Sf*9 cells were infected with the indicated viruses at an MOI of 0.5. The cell culture supernatants were harvested at intervals of 24 h, and the viral titers were determined by an endpoint dilution assay in triplicate.

To investigate the impact of the phosphorylation modification of LEF-6 on late gene expression, we generated another panel of recombinant baculoviruses in which the expression of LEF-6 dephosphorylated mutants was driven by its native promoter; meanwhile, a GFP as a late gene reporter could be produced under the control of *p10* promoter (as illustrated in Fig. 1A). In *Sf*9 cells infected by the baculoviruses, mutations of Y73A, S105A, S108A, Y114A, S119A, S156A, and S159A did not result in significant differences in the GFP fluorescence. Only S95A and 8A mutants resulted in a severe reduction of the GFP fluorescence intensity (Fig. 7A). The fluorescence microscopy results were confirmed by Western blot analysis, which showed that all LEF-6 mutant proteins, especially 8A, were expressed at high levels. Thus, the reduction in GFP expression was not due to lower levels of expression of the different LEF-6 variants (Fig. 7B). To confirm the role of S95 phosphorylation in the viral late gene expression regulation, an S95E mutant containing the mutation of serine 95 to glutamic acid was constructed to mimic the phosphorylation modification at this site. By fluorescence microscopy (Fig. 7A) and Western blot (Fig. 7C) analyses, it turned out that the simulated phosphorylation at the amino acid site 95 restored the function of LEF-6 in promoting viral late gene expression. The growth curves of the baculovirus mutants revealed that the dephosphorylated mutation of S95A on LEF-6 delayed the virus production at 2 and 3 dpi compared with the WT LEF-6, while the simulated phosphorylation mutant S95E showed similar replication ability with its parental virus at 3 dpi (Fig. 7D).

**Fig 8:**
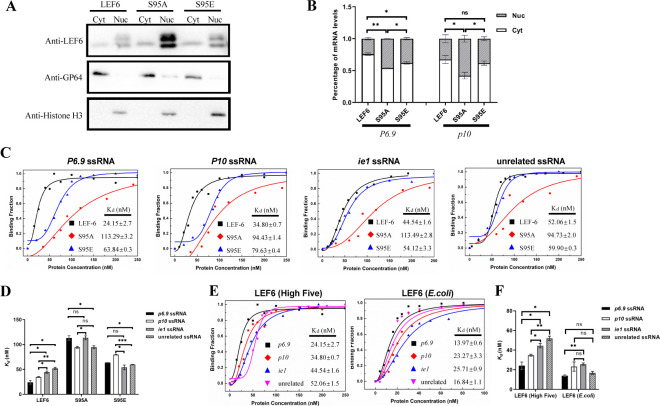
Phosphorylation at S95 affects the specific binding and trafficking of viral RNAs by LEF-6. (**A**) Detection of the protein levels in the fractionated cytoplasmic and nuclear samples by Western blot. GP64 was detected as a reference protein in the cytoplasmic fraction and Histone H3 as a nuclear protein. (**B**) Examination of the viral mRNA distribution between the cytoplasm and nucleus by RT-qPCR. Data were presented as the means ± SD of two replicates. (**C–F**) Determination of the RNA-binding properties of the indicated proteins to ssRNAs by fluorescence anisotropy measurements. The binding curve and *K_d_* values calculation were performed by using Magellan 7 software. The data shown in (**D and F**) were presented as the means ± SD of two replicates. ns: not significant, *P  <  0.05, **P  <  0.01, ***P  <  0.001.

Using nuclear-cytoplasmic fractionation, we investigated whether the mutation of S95 influenced the viral RNA trafficking property of LEF-6. Western blot analyses revealed that the LEF6_S95A_ mutant was substantially accumulated in the nucleus of the virus-infected *Sf*9 cells (Fig. 8A). Accordingly, the proportions of *p6.9* and *p10* mRNAs retained in the nucleus were significantly higher in the cells expressing LEF6_S95A_ than the cells producing LEF-6. Simulated phosphorylation mutant LEF6_S95E_ partially restored the viral mRNA trafficking ability of the protein (Fig. 8B).

To further determine the impact of the protein phosphorylation on its RNA-binding property, we compared the RNA-binding affinity of LEF-6 with the S95 mutants by fluorescence anisotropy measurements. The results showed that the S95A mutant had obviously lower binding affinity to all the tested ssRNAs compared to LEF-6. More importantly, it lost the advantage of having higher binding affinity to the viral late gene RNAs than the viral early gene RNA and unrelated RNA. Simulated phosphorylation of the S95E mutant partially recovered the protein-RNA binding affinity, but it did not restore the protein property that can preferentially bind to the viral late gene RNAs (Fig. 8C and D). To confirm the involvement of phosphorylation modification of LEF-6 in its viral RNA-binding property, we purified phosphorylated LEF-6 from High Five cells and non-phosphorylated LEF-6 from *E. coli* cells and compared their RNA-binding abilities. Similar to the truncated LEF6_1-90_ (Fig. 4D and E), the non-phosphorylated LEF-6 had a strong binding ability to all the four ssRNAs but lost its preference to the viral late ssRNAs, especially for the *p10* ssRNA (Fig. 8E and F).

Taken together, these findings suggested that phosphorylation of LEF-6, especially the modification on S95, plays an important role in its specific binding and trafficking of viral late transcripts into the cytoplasm to promote the translation of these viral proteins in baculovirus infection.

## DISCUSSION

The *lef-6* gene can accelerate the transcription of viral late and very late genes of AcMNPV, although it is nonessential for viral DNA replication or late gene transcription (10). However, it remains unknown how LEF-6 regulates late transcription in baculovirus infection. In this study, we confirmed that *lef-6* was not essential for AcMNPV, but its deletion substantially reduced BV production. Using GFP as a reporter protein, we found that the protein production under the control of viral late *p6.9* promoter and very late *p10* and *ph* promoters dramatically declined after the deletion of *lef-6*. It is noteworthy that the LEF-6 protein level produced by our REP virus was much lower than the WT virus, but it seemed that the low level of LEF-6 was enough to repair its function in promoting viral late protein expression and BV production. In the REP virus, we used the upstream 105 bp fragment in the *lef-6* gene as its native promoter based on the transcriptome data from a previous report (2). This fragment is much shorter than the 875 bp promoter chosen by another study (10). As the expression level of LEF-6 driven by the 875 bp promoter was not examined in the previous report (10), further studies are needed to determine whether the longer promoter can fully restore the expression level of LEF-6 and whether any expression-enhancing elements exist in the upstream sequences or close to the gene locus of the *lef-6* core promoter.

By nuclease digestion of the LEF-6 protein purified from AcMNPV-infected insect cells, we identified LEF-6 as an RNA-binding protein (RBP). RNA sequencing analysis of the RNAs co-purified with LEF-6 suggested that the protein could preferentially interact with viral late and very late transcripts (*p6.9* and *p10* mRNAs), which is consistent with its function in accelerating viral late gene expression. RBPs play many important roles in RNA-related bioprocesses, such as the regulation of RNA stability, transcription, splicing, transportation, and translation by binding to RNA molecules. RBPs typically contain one or more RNA-binding domains (RBDs), which interact with RNA molecules through hydrogen bonds, van der Waals interactions, salt bonding, π-interactions, aromatic stacking, and hydrophobic interactions. The RBD is often a domain of approximately 90 residues, containing two conserved RNPs that are usually separated by 25–35 residues (32). RNA recognition motifs (RRMs) in RBDs commonly adopt a β1α1β2β3α2β4 topology, forming two α-helices against an antiparallel β-sheet, and the conserved RNP1 and RNP2 motifs are located in the β1 and β3 strands (33).

Predicted structure of LEF-6 shows that the protein only has three β-sheets, and the two β-sheets containing the predicted ribonucleoprotein (RNP) motifs may play the RNA-binding role. Unfortunately, the roles of the candidate RNPs in LEF-6 were not verified experimentally in this study, as mutations in the RNPs severely impaired the protein production. However, our data confirmed that the N-terminal half of LEF-6, containing the predicted RNPs, functioned as the RBD. Our studies reveal that LEF-6 has an RNA-binding domain at the N-terminus and an acid-rich domain at the C-terminus, which is similar to the overall structure of heterogeneous nuclear ribonucleoprotein (hnRNPC) with only one RNA-binding domain and an acid-rich auxiliary domain at the N- or C-terminal side of the RBD (34). However, as the RBD, LEF6_1-90_ could interact with both viral and unrelated RNA molecules with high affinity, but the expression of LEF6_1-90_ could not rescue the function of LEF-6 in accelerating viral late gene expression and BV production.

Based on the finding that the coding region of LEF-6 showed more than 80% probability of being related to TAP (3), a member of nuclear mRNA export factor, we examined whether LEF-6 participated in the nuclear export of viral mRNAs after transcription. Using nuclear-cytoplasmic fractionation, we found that most *p10* and *p6.9* mRNAs were readily transported into the cytoplasm in the presence of LEF-6 at 36 hpi. In contrast, more than 70% of the mRNAs were retained in the nucleus when only the LEF6_1-90_ fragment was expressed. These data confirmed the involvement of LEF-6 in viral mRNA trafficking and also highlighted the requirement of the LEF-6 C-terminal domain for its RNA transport function as well as its viral RNA-binding specificity.

Nuclear mRNA-binding proteins usually possess a nuclear localization signal (NLS), which allows the proteins to enter the nucleus. These proteins also frequently undergo post-translational modifications, such as methylation, phosphorylation, and ubiquitination, leading to their changes in subcellular localization and biological activity (35). In the C-terminal half of LEF-6, we identified an NLS motif of KRPRR. Deletion of this motif resulted in the retention of LEF-6 in the cytoplasm and reduced viral mRNA export in the nucleus. We also found that AcMNPV LEF-6 was highly phosphorylated, and seven of its eight phosphorylated sites were located in the C-terminal domain. By site-directed mutagenesis, the phosphorylation of S95 was determined as a key modification site that affected the protein function. Dephosphorylation at S95 resulted in declined viral RNA-binding affinity and specificity, accumulation of the protein mutant in the nucleus, delayed export of *p6.9* and *p10* mRNAs, reduced expression of viral late proteins, and delayed BV production.

To understand how the phosphorylation modification of LEF-6 affects its binding to different RNA fragments, we predicted the complex structure of phosphorylated and non-phosphorylated LEF-6 with the baculovirus *p10*, *p6.9*, and *ie1* ssRNAs using AlphaFold3 (28) (https://golgi.sandbox.google.com). Structure prediction shows that the C-terminal half of non-phosphorylated LEF-6 has a loose structure, while the N-terminal RBD is well exposed, allowing close contact between the RBD and all the three ssRNAs. Phosphorylation modification of LEF-6 at multiple sites can benefit the folding of the C-terminal acid-rich domain and its interaction with the N-terminal RNA-binding domain and selectively allow some RNA strands (such as *p10* and *p6.9* ssRNAs, but not *ie1* ssRNA) to access the RBD. Dephosphorylation mutation at S95 causes the C- and N-terminal ends of the protein to come into close contact, preventing the direct binding of the RBD to RNA molecules (Fig. S11). These predicted structures are consistent with the RNA-protein binding data: non-phosphorylated LEF-6 binds to all different ssRNAs with high affinity, phosphorylated LEF-6 binds to baculovirus late gene *p6.9* and very late gene *p10* ssRNAs with higher affinity than the early gene *ie1* ssRNA, and S95A mutant has decreased binding ability to all the oligonucleotides.

Phosphorylation is an important post-translational modification of proteins that can modulate biological functions involved in signal transduction, cell apoptosis, protein synthesis, subcellular localization, protein-protein interactions, and binding activity (21, 36). Many phosphorylated proteins have been found in baculovirus-encoded proteins, and their phosphorylation plays important roles in the life cycle of baculoviruses. The virus-encoded protein serine/threonine kinase (PK1) mediates hyperphosphorylation of P6.9, which is a prerequisite for high expression of very late genes (37). It remains to be investigated whether the phosphorylation of LEF-6 is catalyzed by the viral phosphokinase PK1. It is noteworthy that in this study, we show that phosphorylated LEF-6 can preferentially bind and export baculovirus late and very late RNAs, including *p6.9* mRNA. As a late transcription regulator (37, 38), the upregulation of P6.9 expression could further enhance the very late gene transcription.

In conclusion, we identified AcMNPV LEF-6 protein as an RNA-binding protein involved in the nuclear export of viral late transcripts. The phosphorylation modifications, especially the phosphorylation at the amino acid site 95, are required for LEF-6 to gain its viral RNA-binding specificity and complete its mRNA transport function. Our study explains how LEF-6 promotes the production of viral late proteins and consequently accelerates the AcMNPV infection cycle.

## Data Availability

All unique reagents generated in this study are available from the lead contact without restriction. The raw sequence data for the LEF-6 bound RNAs have been deposited in the Genome Sequence Archive (39) in National Genomics Data Center (40), China National Center for Bioinformation / Beijing Institute of Genomics, Chinese Academy of Sciences (GSA: CRA020718).

## SUPPLEMENTAL MATERIAL

Supplemental material jvi.02100-24-s0001.pdf Figures S1 to S11; Tables S1 and S2.

ASM does not own the copyrights to Supplemental Material that may be linked to, or accessed through, an article. The authors have granted ASM a non-exclusive, world-wide license to publish the Supplemental Material files. Please contact the corresponding author directly for reuse.
