# Supplementary material for: LEF-6 phosphorylation regulates the binding and trafficking of baculovirus late gene transcripts
Source: J Virol. 2025 Aug 11;99(9):e02100-24. doi: 10.1128/jvi.02100-24 (PMC12455930; doi:10.1128/jvi.02100-24)
Supplement: Supplemental material — Figures S1 to S11; Tables S1 and S2. [file jvi.02100-24-s0001.pdf]

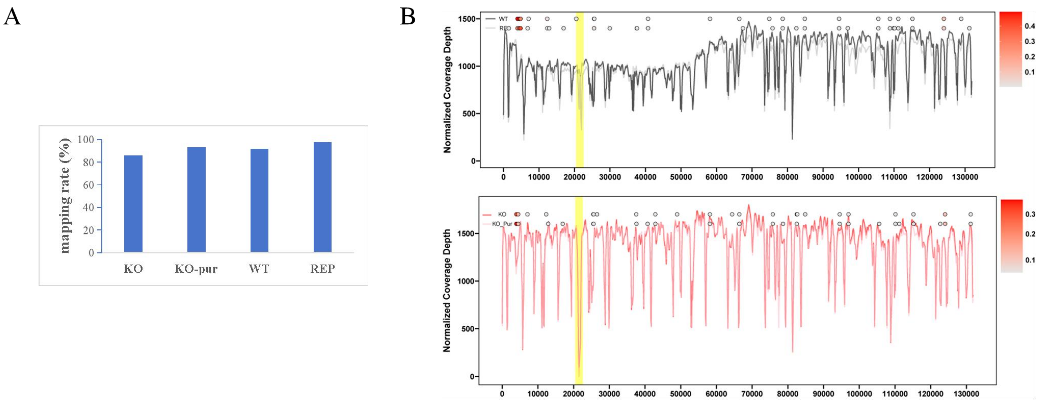

Figure S1. Examination of the homogeneity of baculoviruses generated by homologous recombination. A. Percentage of sequencing reads mapped to AcMNPV reference genome. B. Sequencing reads mapped to the reference virus genome. The yellow-shaded region highlights the lef6 locus. The circles above the curves indicate the mutation sites, for which the detailed information is listed in table S2. WT: Control virus (vAc) generated by co-transfection of *Sf*9 cells with bAc and pBac5/p10p-GFP. KO: vAc∆lef6. KO-pur: vAc∆lef6 amplified from cloned virus picked by plaque purification. REP: vAc∆lef6/lef6p-lef6.

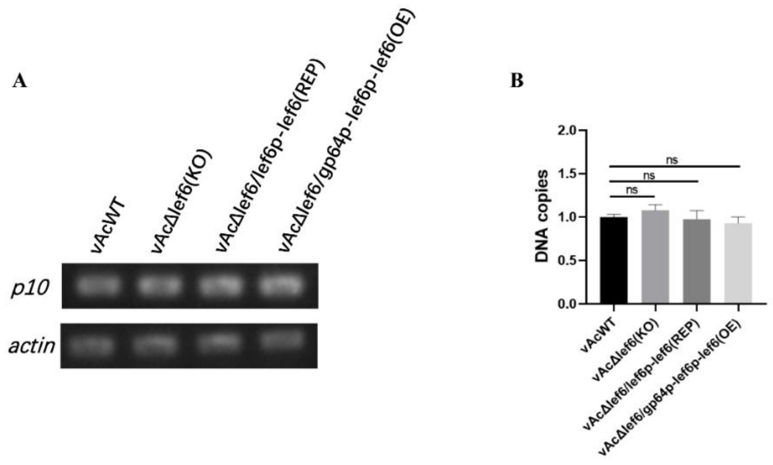

Figure S2. Examination of viral DNA replication. A. Detection of viral DNA by PCR. B. Quantification of viral DNA by real time quantitative PCR. *Sf*9 cells were

infected with the indicated viruses at an MOI of 3. Total DNA was extracted from the infected cells at 24 hpi. The *p10* gene fragment was amplified to represent viral DNA, and cellular *β-actin* gene fragment was amplified as a control. Data are presented as the means ±SD of three replicates. ns: not significant.

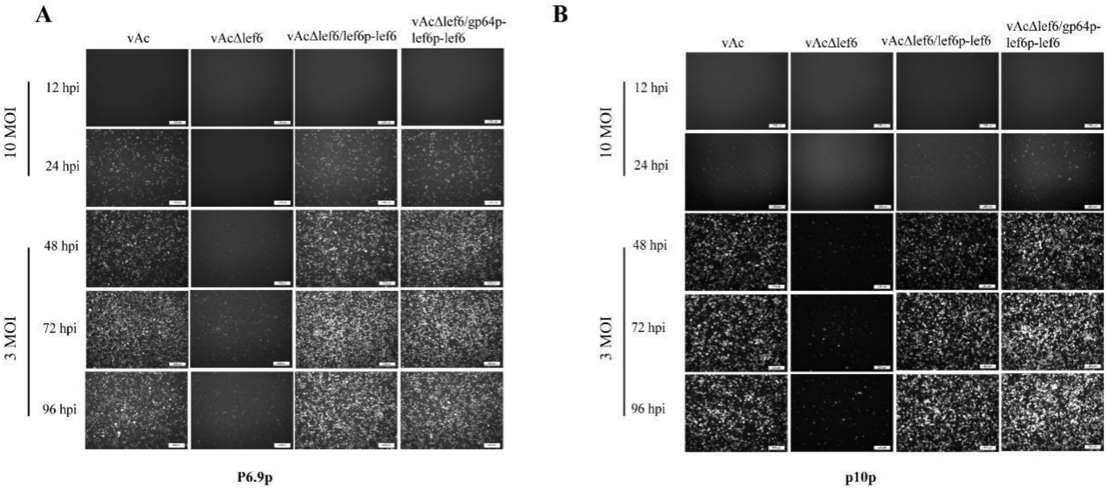

Figure S3. Observation of reporter GFP expression by fluorescence microscopy. The GFP expression was controlled by baculovirus late gene *p6.9* or very late gene *p10* promoters. *Sf*9 cells were infected with the indicated recombinant viruses at an MOI of 3 or 10. The images were taken at 2, 3 and 4 dpi for the cells infected at 3 MOI, and at 12 and 24 hpi for the cells infected at 10 MOI.

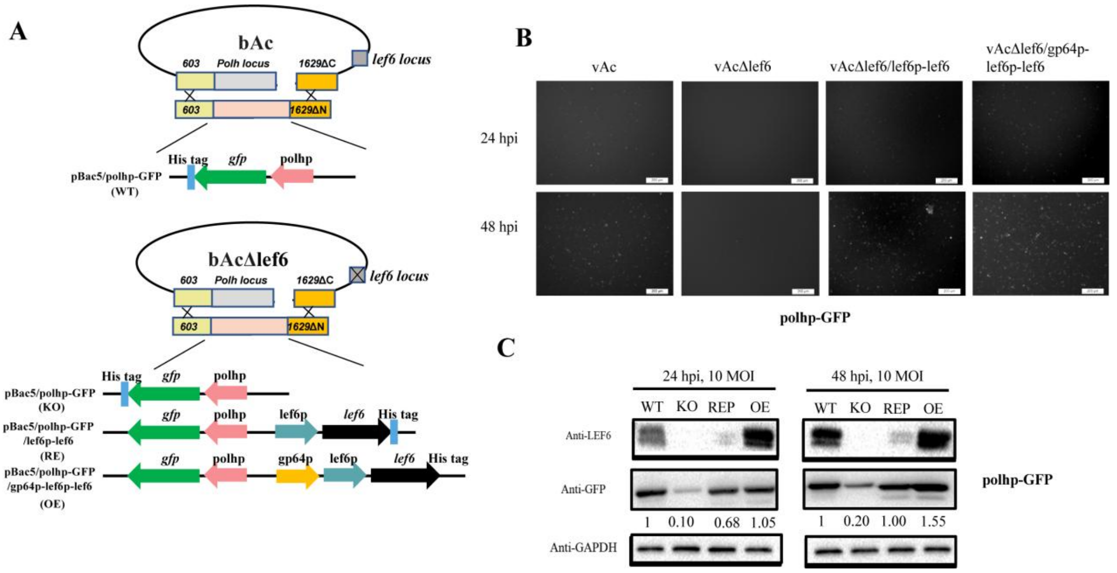

Figure S4. Effects of deletion and complementation of *lef-6* on GFP expression driven by *polyhedrin* promoter. A. Schematic diagram of the generation of wild type (vAc, WT), *lef-6* KO (vAcΔlef6, KO), *lef-6* restored (vAcΔlef6/lef6p-lef6, REP) and *lef-6* overexpression (vAcΔlef6/gp64p-lef6p-lef6, OE) viruses which express GFP under the control of AcMNPV very late *ph* promoter. B. Observation of reporter GFP expression by fluorescence microscopy. *Sf*9 cells were infected with the indicated viruses at an MOI of 10, and the GFP expression was observed at 24 and 48 hpi. C. Detection of GFP expression by Western blot. Total cell lysates were separated by SDS-PAGE and respectively probed by anti-LEF6, anti-GFP and anti-GAPDH antibody. GAPDH was detected as a loading control. The GFP bands were quantified by densitometry scanning using Image J software, and the relative expression levels to the WT are shown below the GFP bands.

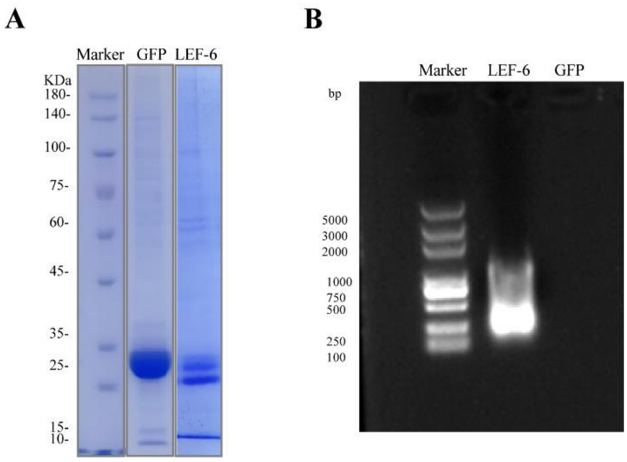

Figure S5. Detection of nucleic acids co-purified with LEF-6 protein. A. Examination of His-tagged LEF-6 protein purified from High Five cells by SDS-PAGE. His-tagged GFP was purified from High Five cells infected with vAc/p10p-GFP (Fig.1A） and used as a control protein. B. Detection of the nucleic acids co-purified with LEF-6 by agarose gel electrophoresis. No nucleic acid band was detected from purified GFP.

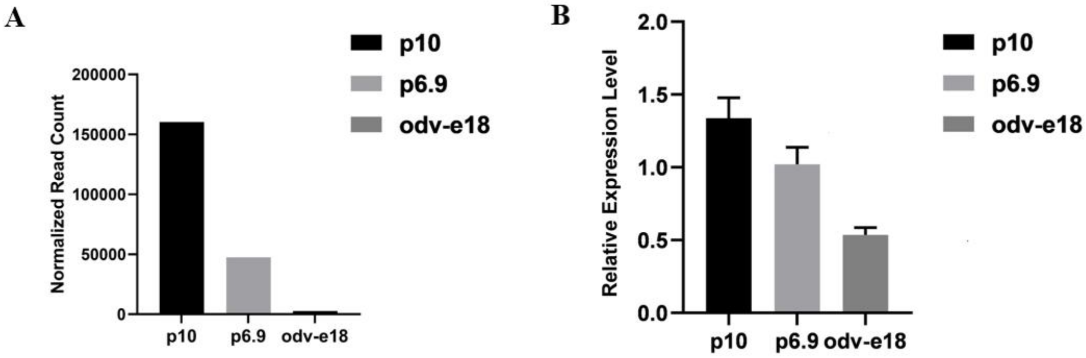

Figure S6. Comparison of the relative mRNA levels of *p10*, *p6.9* and *odv-e18* expressed in AcMNPV-infected cells and co-purified with LEF-6. A. The mRNA reads determined by RNA-seq. LEF-6 was purified from AcMNPV-infected High Five cells at 3 dpi, and the RNAs bound with purified LEF-6 were determined by RNA sequencing. B. The relative mRNA levels quantified by real time RT-PCR in

AcMNPV-infected High Five cells at 3 dpi.

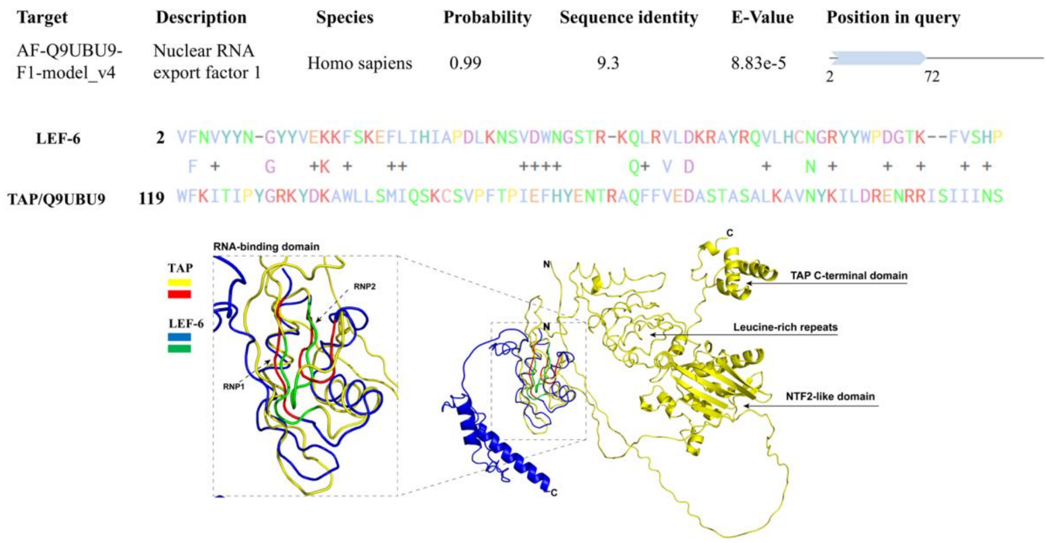

Figure S7. Structure alignment and superposition of LEF-6 and TAP performed by using Foldseek program. The alignment of the amino acid sequences for the RNA binding domains of the two proteins are shown above the 3D structures of the proteins.

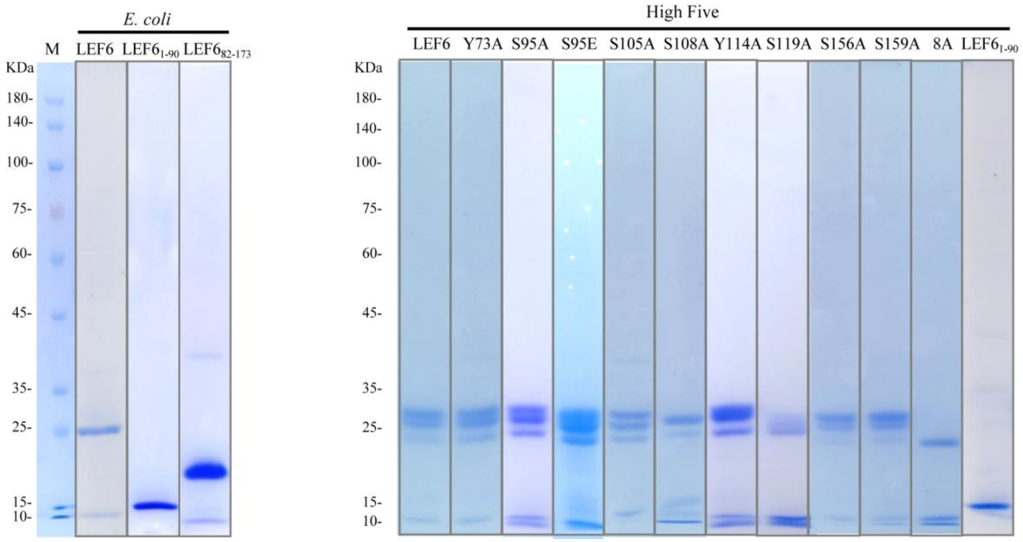

Figure S8. Purified LEF-6 and its mutants produced in this study. Left panel,

proteins purified from *E. coli* cells. Right panel, proteins purified from High Five insect cells.

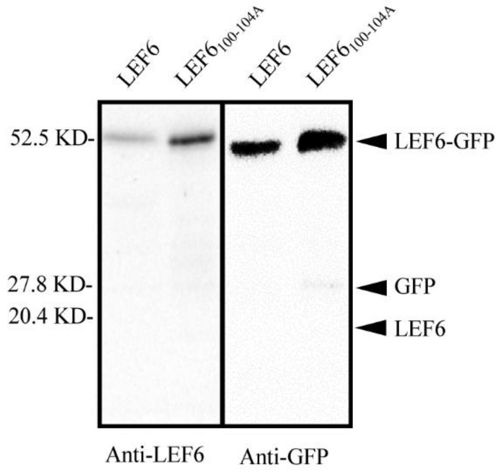

Figure S9. Examination of the integrity of LEF6-GFP fusion proteins by Western blot. *Sf*9 cells were transfected with the plasmids shown in Fig. 5A to express GFP-tagged LEF-6 and $LEF6_{100-104A}$, and the expression of GFP-fusion proteins observed by fluorescence microscope was shown in Fig. 5B. Lysates of the transfected cells were respectively probed with anti-LEF6 (left panel) and anti-GFP (right panel) antibodies.

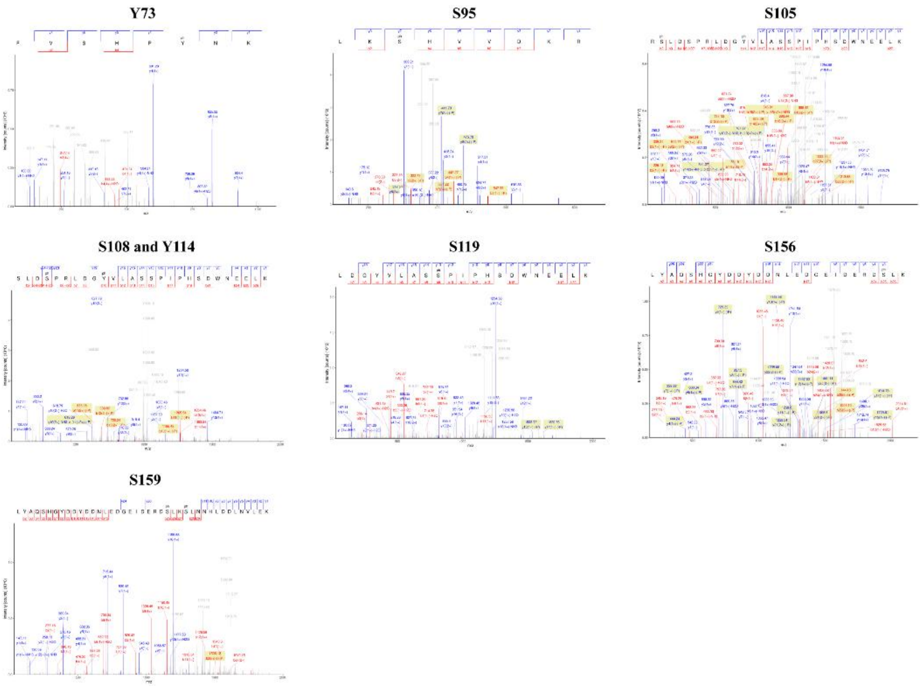

Figure S10. MS/MS spectrum of the LEF-6 phosphorylated peptides. LEF-6 protein was purified from baculovirus infected High Five cells, resolved by SDS-PAGE, digested in-gel and then analyzed by MS/MS. Phosphorylated ion is characterized by a neutral loss of 79.97 Da. The y and b ions are marked in blue and red, respectively.

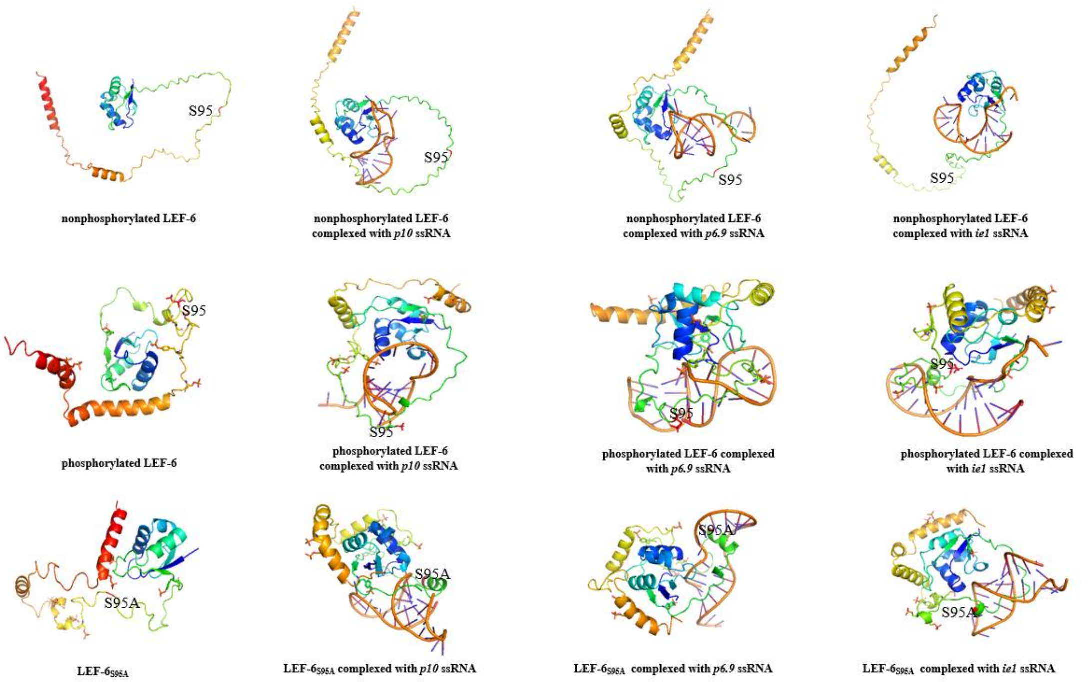

Figure S11. The structures of LEF6-ssRNA complexes predicted by AlphaFold3. RNA strands are shown in orange. Phosphate groups are shown in red sticks. The position of the S95 amino acid in the protein is marked.

Table S1 Primers employed in PCR

Name	Primer sequence (5'-3')
lef6U	gagcaactatatattgatagggcatttttgcgtttgcg
pBac5-lef6-Xhol-R	gctgctcgagttgtttttctaatacattcaagtcgtc
pBac5-GFP-BglII-F	gcagagatctttacttgtacagctcgtccat
pBac5-GFP-R	gccctatcaatatatagttgctctagccgggacctttaattcaac
gp64-F	gcaactatatattgatagccagataaaaataatcttac
pgp64-plef6-R	cgcaaaaatgccgtgtgttccttattgaag
pBac5-GFP-D	ggctatcaatatatagttgctctagccgg
pgp64-plef6-F	aggaacacacggcatttttgcgtttgcg
pp6.9-BglII-F	gccgagatctccggaacaaaaattcaaacgac
pp6.9-NcoI-R	gcggccatggtatatctccttttgtgtaatttatgtagct
OpIE2-lef6-NCOI-F	ttaccatggtggtgttcaacgtgta
OpIE2-lef6-BamHI-R	cggggatccttgtttttctaatac
lef6-100-104A-F	gacgctgcagcggctgcctctttagattctcctcgct
lef6-100-104A-R	ggcagccgctgcagcgtcgaccacgtggctttttaatcg
lef6-RNP2A-F	gctgcagcggctgccgcaggctattatgtggaaaaaaaattc
lef6-RNP2A-R	tgcggcagccgctgcagccaccattttattacacca
lef6-RNP1A-F	gctgctgcagcagctgccgctgcacgcgcctacaggcaggtgttgcactgcaa
lef6-RNP1A-R	ggcagctgctgcagcagccgcagctgcgccgttccagtcgacgctgtttttcaaatc
lef6-1-91-XhoI-R	taatctcgagcgagctgtcggtccgttt
Y73A-F	ctctcatccggccaacaaatctattcgc
Y73A-R	tagatttgttggccggatgagagacaaa
S95A-F	tcgattaaaagcacacgtggtcgacaaa
S95A-R	cgaccacgtgtgcttttaatcgatgcga
S95E-F	tcgattaaaagagcacgtggtcgacaaa
S95E-R	cgaccacgtgctcttttaatcgatgcga
S105A-F	aaacgaccgcgccgcgctttagattctcctcgc
S105A-R	gcgaggagaatctaaagcgcggcgcggtcgttt
S108A-F	gctctttagatgctcctcgcttggacggat
S108A-R	aagcgaggagcatctaaagagcggcgcgg
Y114A-F	cttggacggagctgttttggcatcgtcgcc
Y114A-R	atgccaaaacagctccgtccaagcgagg
S119A-F	tatgttttggcatcggcgcccataccacacagc

Name	Primer sequence (5'-3')
S119A-R	gctgtgtggtatgggcgccgatgccaaaacata
S156A-F	cgaacgtgacgctttaaaaagtttaaata
S156A-R	ctttttaaagcgtcacgttcgtcgatttc
S159A-F	cgtgactctttaaaagctttaaataatcatcta
S159A-R	tagatgattatttaaagcttttaaagagtcacg
pTriEx-NcoI-F	gcatccatggtgttcaacgtgtactaca
pTriEx-1-90-R	taatctcgagcgagctgtcggtccgttt
pTriEx-82-173-F-	gtatccatggcaacagtcaaacggacc
pTriEx-XhoI-R	cgcgctcgagttgtttttctaatacattc
Ac28U	cgacgtgtcgtcaggacgtcaccgattttataaaaatatttgtggtgtaataaagatggcctggtgatgg
Ac28D	cattatatattacaaaatatagttttattattattatttttaatacatgttttattaccaatgcttaatc
Ac28UT	aacgccgacgacattgag
1st UT	cacacaatatgaggacgc
Ac28F	cgacgtgtcgtcaggacgtcaccgattttataaaaatatttgtggtgtaataaataaaacat
Ac28R	cattatatattacaaaatatagttttattattattatttttaatacatgttttatttattac
Ac28DT	aagtcaagacaaaactttc
p6.9-qPCR-F	agcgtgttctgtaacttcgg
p6.9-qPCR-R	accacatatggttcgacacg
p10-qPCR-F	ccgctcaattgaccgatctt
p10-qPCR-R	gcttggcttttcagcttagg
odv-e18-qPCR-F	gttcttgaccatcttggctgtag
odv-e18-qPCR-R	gctcgcatggtagcgtttaaagg

Table S2 Details for the mutation sites detected in virus stocks by deep sequencing

**Virus**	**Position in reference Bacmid**	**Ref**	**Alt**	**Type**	**Ref_depth**	**Alt_depth**	**Total_depth**	**Mut_Freq**
KO	3961	T	C	synonymous	170	78	248	0.314516129
KO	4478	aggggggggggggg	agggggggggggg, aggggggggggg	intergenic	123	37	160	0.23125
KO	12163	C	T	synonymous	184	24	208	0.115384615
KO	14757	A	T	ND	246	1	247	0.004048583
KO	20013	A	G	ND	174	2	176	0.011363636
KO	33198	C	T	ND	229	2	231	0.008658009
KO	33264	G	T	ND	246	1	247	0.004048583
KO	34246	A	T	ND	244	1	245	0.004081633
KO	45223	T	G	ND	246	1	247	0.004048583
KO	50568	C	T	ND	241	1	242	0.004132231
KO	56665	A	T	ND	244	1	245	0.004081633
KO	65854	G	A	ND	245	1	246	0.004065041
KO	72077	T	G	ND	245	1	246	0.004065041
KO	74102	A	C	ND	251	2	253	0.007905138
KO	83469	T	C	ND	243	1	244	0.004098361
KO	90173	T	A	ND	245	1	246	0.004065041
KO	90324	G	T	ND	245	1	246	0.004065041
KO	92539	T	G	ND	233	2	235	0.008510638
KO	102195	T	G	ND	232	1	233	0.004291845
KO	104652	T	A	ND	248	1	249	0.004016064
KO	117822	C	A	ND	250	1	251	0.003984064
KO	122869	T	C	ND	246	1	247	0.004048583
KO	131721	tcgccgccgccg	tcgccgccg	ND	223	20	243	0.082304527
KO	138889	A	G	ND	239	1	240	0.004166667
KO_Pur	3961	T	C	synonymous	155	92	247	0.372469636
KO_Pur	4478	aggggggggggggg	agggggggggggg, aggggggggggg	intergenic	106	30	136	0.220588235
KO_Pur	12163	C	T	synonymous	151	15	166	0.090361446
KO_Pur	20556	T	A	ND	247	1	248	0.004032258
KO_Pur	24623	T	G	ND	246	1	247	0.004048583
KO_Pur	33198	C	T	ND	234	5	239	0.020920502
KO_Pur	33264	G	T	ND	248	1	249	0.004016064
KO_Pur	45283	A	G	ND	245	1	246	0.004065041
KO_Pur	48395	A	C	ND	246	1	247	0.004048583
KO_Pur	50568	C	T	ND	239	1	240	0.004166667
KO_Pur	65854	G	A	ND	245	1	246	0.004065041
KO_Pur	74085	A	G	ND	247	3	250	0.012
KO_Pur	74102	A	C	ND	253	2	255	0.007843137

Virus	Position in reference Bacmid	Ref	Alt	Type	Ref_depth	Alt_depth	Total_depth	Mut_Freq
KO_Pur	83469	T	C	ND	238	1	239	0.0041841
KO_Pur	86325	C	T	ND	245	2	247	0.008097166
KO_Pur	90173	T	A	ND	246	1	247	0.004048583
KO_Pur	102195	T	G	ND	234	1	235	0.004255319
KO_Pur	104643	A	T	ND	242	1	243	0.004115226
KO_Pur	113250	A	G	ND	240	1	241	0.004149378
KO_Pur	117822	C	A	ND	245	1	246	0.004065041
KO_Pur	118874	C	T	ND	243	3	246	0.012195122
KO_Pur	122869	T	C	ND	241	2	243	0.008230453
KO_Pur	130372	C	T	ND	243	1	244	0.004098361
KO_Pur	131721	tcgccgccgccg	tcgccgccg	ND	223	22	245	0.089795918
KO_Pur	138889	A	G	ND	234	1	235	0.004255319
REP	1496	T	A	ND	247	1	248	0.004032258
REP	3961	T	C	synonymous	183	66	249	0.265060241
REP	4478	agggggggggggg	aggggggggggg	intergenic	203	40	243	0.164609053
REP	12163	C	T	synonymous	151	93	244	0.381147541
REP	12495	G	A	synonymous	144	105	249	0.421686747
REP	14510	T	A	ND	246	1	247	0.004048583
REP	20013	A	G	ND	197	2	199	0.010050251
REP	20556	T	A	ND	246	1	247	0.004048583
REP	24623	T	G	ND	246	1	247	0.004048583
REP	33198	C	T	ND	219	7	226	0.030973451
REP	37630	T	C	ND	245	1	246	0.004065041
REP	45223	T	G	ND	245	1	246	0.004065041
REP	45283	A	G	ND	241	4	245	0.016326531
REP	48395	A	C	ND	246	1	247	0.004048583
REP	75131	T	A	ND	246	1	247	0.004048583
REP	83469	T	C	ND	244	2	246	0.008130081
REP	86325	C	T	ND	240	2	242	0.008264463
REP	90173	T	A	ND	244	1	245	0.004081633
REP	92539	T	G	ND	237	4	241	0.01659751
REP	102195	T	G	ND	233	4	237	0.016877637
REP	104652	T	A	ND	243	1	244	0.004098361
REP	113250	A	G	ND	244	2	246	0.008130081
REP	116536	T	A	ND	236	1	237	0.004219409
REP	117596	A	T	ND	243	1	244	0.004098361
REP	117822	C	A	ND	251	1	252	0.003968254
REP	118874	C	T	ND	244	3	247	0.012145749
REP	122869	T	C	ND	241	2	243	0.008230453
REP	131721	tcgccgccgccg	tcgccgccg	ND	222	18	240	0.075
REP	138889	A	G	ND	245	1	246	0.004065041

Virus	Position in reference Bacmid	Ref	Alt	Type	Ref_depth	Alt_depth	Total_depth	Mut_Freq
WT	3961	T	C	synonymous	126	121	247	0.489878543
WT	12163	C	T	synonymous	126	122	248	0.491935484
WT	12495	G	A	synonymous	177	71	248	0.286290323
WT	14757	A	T	ND	246	1	247	0.004048583
WT	20013	A	G	ND	173	4	177	0.02259887
WT	28180	G	A	ND	244	1	245	0.004081633
WT	33198	C	T	ND	245	1	246	0.004065041
WT	33264	G	T	ND	246	1	247	0.004048583
WT	48395	A	C	ND	231	1	232	0.004310345
WT	65854	G	A	ND	246	1	247	0.004048583
WT	74102	A	C	ND	248	1	249	0.004016064
WT	82533	A	C	ND	242	1	243	0.004115226
WT	86325	C	T	ND	245	2	247	0.008097166
WT	92539	T	G	ND	240	1	241	0.004149378
WT	102195	T	G	ND	244	1	245	0.004081633
WT	113250	A	G	ND	244	3	247	0.012145749
WT	116536	T	A	ND	242	1	243	0.004115226
WT	118874	C	T	ND	245	2	247	0.008097166
WT	122869	T	C	ND	241	3	244	0.012295082
WT	131721	tcgccgccgccg	tcgccgccg	ND	220	24	244	0.098360656
WT	136598	A	T	ND	242	1	243	0.004115226
